# Genomic Insights and Synthetic Biology Applications of Marine Actinomycete *Streptomyces griseoincarnatus* HNS054

**DOI:** 10.3390/ijms25063127

**Published:** 2024-03-08

**Authors:** Qinghua Wang, Jing Zhao, Zhaoyuan Liu, Shaoxiong Ding, Zhiyong Huang, Jun Chen

**Affiliations:** 1 State Key Laboratory of Marine Environmental Science, Xiamen University, Xiamen 361102, China; wangqinghua@stu.xmu.edu.cn (Q.W.); sunnyzhaoj@xmu.edu.cn (J.Z.); liuzhaoyuan2919@163.com (Z.L.); sxding@xmu.edu.cn (S.D.); 2 Department of Marine Biological Science and Technology, College of Ocean and Earth Sciences, Xiamen University, Xiamen 361102, China; 3 State-Province Joint Engineering Laboratory of Marine Bioproducts and Technology, Xiamen University, Xiamen 361102, China; 4 Tianjin Institute of Industrial Biotechnology, Chinese Academy of Sciences, Tianjin 300308, China; huang_zy@tib.cas.cn

**Keywords:** marine actinomycete, chassis, natural product, biosynthetic gene cluster, rapid growth, MSGE

## Abstract

The marine bacterium *Streptomyces* sp. HNS054 shows promise as a platform for producing natural products. Isolated from a marine sponge, HNS054 possesses several desirable traits for bioengineering: rapid growth, salt tolerance, and compatibility with genetic tools. Its genome contains 21 potential biosynthetic gene clusters, offering a rich source of natural products. We successfully engineered HNS054 to increase the production of aborycin and actinorhodin by 4.5-fold and 1.2-fold, respectively, compared to *S. coelicolor* M1346 counterparts. With its unique features and amenability to genetic manipulation, HNS054 emerges as a promising candidate for developing novel marine-derived drugs and other valuable compounds.

## 1. Introduction

Recent advances in genome sequencing, especially marine microbial metagenomics, have amassed a rich wealth of marine biosynthetic gene clusters (BGCs) [1]. These BGCs represent a valuable resource for drug discovery and development, but they need to be expressed in suitable host cells. Host cells should be able to perform complex biosynthetic pathways and tolerate possible toxic effects of heterologous products. Actinomycetes serve as excellent hosts for expressing a wide range of bioactive compounds, including antibiotics, antitumor agents, immunosuppressants, and pigments. Their versatility makes them ideal for overexpressing complex and biotoxic natural products, finding applications across diverse sectors such as medicine, agriculture, food, and industry [2,3].

Noteworthy examples such as *S. coelicolor* [4], *S. lividans* [5,6], *S. albus* [7], and *S. avermitilis* [8], which originate from terrestrial environments, serve as mature platforms for the heterologous expression of various complex natural products. However, marine natural products may have different structures and activities than terrestrial ones, and marine actinomycetes may be more compatible with them. Nevertheless, progress in the development of marine actinomycete chassis has been relatively constrained. To date, only two instances stand out: the establishment of *Salinispora tropica* CNB-4401 [9] and MGCEP 1.0 [10], derived from the marine actinomycetes *S. tropica* CNB-440 and *S. atratus* SCSIO ZH16, respectively.

Actinomycete chassis cells undergo diverse genetic enhancements, involving genome simplification [11], removal of native secondary metabolite BGCs [12], insertion of site-specific recombination (SSR) system *attB* loci to integrate multiple foreign BGCs [13], and removal of negative regulators of BGC expression [14,15]. These modifications aim to enhance the heterologous expression of foreign BGCs [16]. Consequently, potential hosts earmarked as chassis should be compatible with genetic engineering tools.

In our prior investigations, *Streptomyces* sp. HNS054, isolated from a marine sponge *Mycale* sp. [17], underwent extensive examination for its proficiency in novel natural product synthesis [18]. HNS054 exhibits robust growth and shows promising signs of being amenable to genetic modification. In this study, the complete genome of HNS054 was sequenced, and genetic engineering methods were applied to evaluate its potential as a host for foreign BGC expression. The aborycin and actinorhodin BGCs served as test cases. Salinity tolerance, growth rates, biomass accumulation, and antibiotic sensitivity were also scrutinized. The results indicate that hosts derived from HNS054 have the potential to be developed as marine actinomycete chassis.

## 2. Results

### 2.1. Genomic Features and Annotation of HNS054

The complete genome of HNS054 (GenBank: CP139576) consists of the linear chromosome approximately 7.5 Mb in size, with a GC content of 72.3%. It encompasses 6678 putative protein-coding genes, 72 transfer RNA (tRNA) genes, and 18 ribosomal RNA (rRNA) genes. A summary of the genome information for HNS054 is presented in Table S1. Although no CRISPR/Cas systems were detected by CRISPRCasFinder, 24 CRISPR arrays were identified (Table S2).

The COG, GO and KEGG database annotations assigned functional categories to 5121, 4766, and 2380 genes in the genome of HNS054, respectively (Figures S1–S3). Among them, 129 genes were associated with secondary metabolite biosynthesis, transport, and metabolism, indicating the strain’s potential to produce various secondary metabolites. Notably, 1194 genes with unknown functions were identified, warranting further exploration.

### 2.2. Comparative Genome and Pan-Genome Analysis

The genomic study of Streptomyces reveals that essential genes are distributed in the core chromosomal region centered around the replication origin (OriC). The subtelomeric regions at both ends of the linear chromosome, containing genes with lower conservation, are identified as non-essential gene regions [19]. Therefore, the delineation of these genomic regions is crucial for guiding the construction of a genome-minimized chassis. The location of the OriC of HNS054 was identified to be at positions 3,740,805–3,741,749 bp, approximately 19.7 Kb away from the chromosomal center at 3,761,515 bp (Figure S4). The OriC has 22 DnaA boxes and an AT-rich region: ACAAAAAA. Multi-genome alignment analysis revealed that the genome of HNS054 is symmetrical, consisting of a core region spanning about 5.73 Mb and two subtelomeric regions housing the accessory genome (Figure S5). The core region features highly conserved and homologous genes, while the accessory genome encompasses dispensable elements, including 18 genomic islands (GIs) predicted by IslandViewer4 (Figure S6). These GIs are primarily concentrated in the subtelomeric regions, potentially contributing to the strain’s adaptation and diversity.

**Figure 1:**
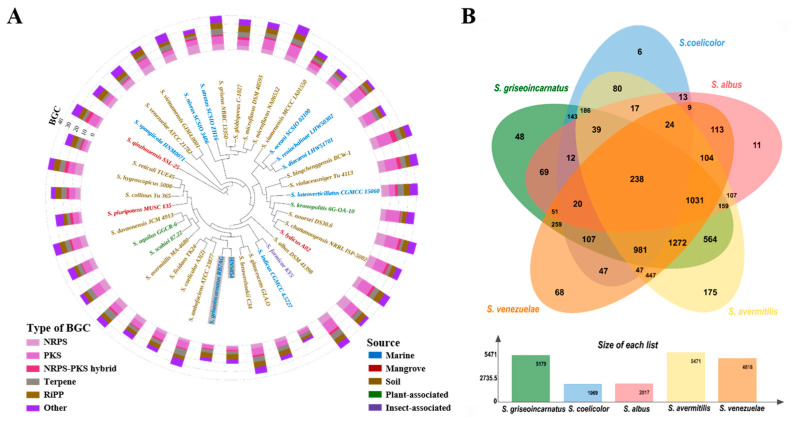
Comparative genomics analysis of strain HNS054. (**A**) Whole-genome-based phylogenetic tree of HNS054 and other Streptomyces. Different strain sources and types of BGC are color-coded. (**B**) Comparison and annotation of orthologous gene clusters among HNS054 (GenBank Accession: CP139576), *S. coelicolor* A3(2) (GenBank Accession: CP042324), *S. albus* DSM 41398 (GenBank Accession: CP010519), *S. avermitilis* MA-4680 (GenBank Accession: NC_003155), and *S. venezuelae* ATCC 21782 (GenBank Accession: CP029190).

Core genome phylogeny revealed a close association between strain HNS054 and *S. griseoincarnatus* RB7AG (Figure 1A). The most recent 16S rRNA data (Figure S7) positioned strain HNS054 within the same cluster as *S. griseoincarnatus* LMG 19316T. Average nucleotide identity (ANI), reflecting the average nucleotide identity of all orthologous genes shared between any two genomes, provided a robust distinction between strains of the same or closely related species, typically showing an 80–100% ANI range. Digital DNA–DNA hybridization (dDDH) values, offering a direct measure of shared gene content weighted by shared sequence similarity in silico, tended to be higher for closer-related species. Both ANI and dDDH data consistently indicated that strain HNS054 was the closest relative to *S. griseoincarnatus* (Table S3). Therefore, HNS054 was confidently classified as *S. griseoincarnatus*.

The core genome phylogeny also shows that HNS054 is closely related to other well-studied Streptomyces chassis strains, such as *S. coelicolor* A3(2) and *S. lividans* TK24 (Figure 1A). This suggests shared evolutionary and metabolic characteristics among these strains. Through multiple genome-wide alignments and comparative genomics analyses, a total of 5179 protein clusters were annotated in HNS054, encompassing 238 highly conserved orthologous gene clusters and 48 clusters unique to this strain (Figure 1B). The functionally enriched proteins in the unique clusters are related to biosynthetic processes, including polysaccharide biosynthesis, ATP binding biosynthesis, DNA-mediated transposition, and plasma membrane biosynthetic processes. These proteins may confer HNS054 strong cell division abilities, leading to rapid growth and high biomass accumulation. The unique clusters also suggest distinctive metabolic capabilities, indicating potential applications in synthetic biology.

### 2.3. Analysis of Secondary Metabolite BGCs

The antiSMASH 7.1.0 predicted 21 BGCs in the genome of HNS054 (Table S4). These BGCs are involved in the synthesis of various types of secondary metabolites, including ribosomally synthesized and post-translationally modified peptides (RiPPs), non-ribosomal peptide synthetases (NRPSs), polyketide synthases (PKSs), NRPS–PKS hybrids, terpenes, ectoines, siderophores, and others. The chromosomal distribution of these BGCs is illustrated in Figure S8. Some of these BGCs have low similarity to known ones, suggesting that HNS054 might produce novel secondary metabolites during fermentation.

### 2.4. Analysis of SSR Systems in Strain HNS054

The genome of HNS054 possesses 10 common-type *attB* sites suitable for eight SSR systems (φC31, φBT1, SV1, TG1, VWB, φK38-1, CBG73463, and φJoe), as depicted in Figure S9 and detailed in Table S5. These *attB* sites exhibit highly conserved core regions, with sequence identities ranging from 89% to 100%. Their distribution spans throughout the genome (Figure S10). SSR systems are powerful tools for Streptomyces’ genetic engineering, facilitating targeted and stable integration of the foreign DNA into the chromosome. HNS054 has multiple *attB* sites, which allow the use of the “multiple integrases multiple *attB* sites” method, for simultaneous multi-site insertion of genes or gene clusters in one step [20]. They also provide a rich resource for various applications, including genome editing, gene expression, plasmid construction, strain engineering, and natural product discovery.

### 2.5. Growth, Salt Tolerance, and Antibiotic Susceptibility of Strain HNS054

**Figure 2:**
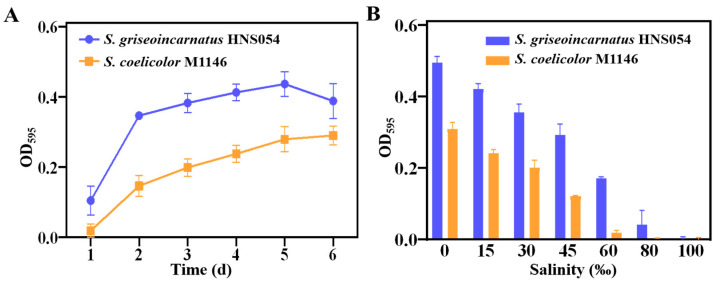
(**A**) Growth curves of HNS054 and *S. coelicolor* M1146 under 30‰ salinity conditions. (**B**) Salt tolerance of HNS054 and *S. coelicolor* M1146.

HNS054 grew faster and had higher biomass compared to *S. coelicolor* M1146 under various salinity conditions (Figure 2A). At 30‰ salinity, the growth rate of HNS054 was 1.8-fold that of M1146. Additionally, the biomass of HNS054 was 1.6-fold that of *S. coelicolor* M1146 during the plateau phase. This suggests that HNS054 possesses a higher salinity optimum or more efficient nutrient utilization or other potential factors than *S. coelicolor* M1146.

HNS054 has a maximum salinity tolerance of 60‰, while *S. coelicolor* M1146 has a maximum salinity tolerance of 45‰ (Figure 2B). The notable high salinity tolerance of HNS054 positions it as an advantageous marine Streptomyces, capable of thriving and producing secondary metabolites in seawater or saline mediums.

The antibiotic sensitivity of HNS054 was tested to find suitable screening markers for genetic engineering. HNS054 was resistant to ampicillin, chloramphenicol, thiostrepton, and nalidixic acid, while displaying sensitivity to kanamycin, apramycin, and tetracycline (Table S6). The minimum inhibitory concentrations (MICs) for kanamycin and apramycin were determined to be 25 μg/mL, and for tetracycline, it was 12.5 μg/mL. Consequently, kanamycin, apramycin, or tetracycline could serve as effective screening markers. For spectinomycin or hygromycin antibiotics, higher concentrations of 200 μg/mL spectinomycin and 100 μg/mL hygromycin were required for screening.

### 2.6. Development of MSGE Hosts from Strain HNS054 Using CRISPR/Cas9 Methodology

Employing the CRISPR/Cas9 methodology, a diverse set of enhanced heterologous hosts was derived from the wild-type HNS054. The initial knockout operation focused on eliminating aborycin production capacity in the wild strain by targeting BGC11. This manipulation resulted in the creation of the HNS1151 host, featuring a single native φC31 *attB* site. Subsequently, strain HNS1251 emerged through the integration of an artificial φC31 *attB* site into the location formerly occupied by BGC11. Expanding on this groundwork, strains HNS1351 to HNS1551 were generated by substituting BGC14, BGC17, and BGC2 with additional artificial φC31 *attB* sites, respectively. Each resulting strain, rigorously validated through PCR and sequencing (Figure S11), had one to five φC31 *attB* sites. A growth analysis showed that strains HNS1151-1551 had similar growth performance to the parent HNS054 (Figure S12).

### 2.7. Improvement of Aborycin Production

The aborycin BGC was integrated into the chromosome of strains HNS1151-1551 using the pSET152::gul plasmid, resulting in strain HNS1151::gul, HNS1251::2gul, HNS1351::3gul, HNS1451::4gul, and HNS1551::5gul (Figure S13). Aborycin production was analyzed through high-resolution HPLC-MS (Figure S14), and a standard curve for aborycin (Figure S15) was utilized to calibrate the aborycin yield based on HPLC peak areas.

**Figure 3:**
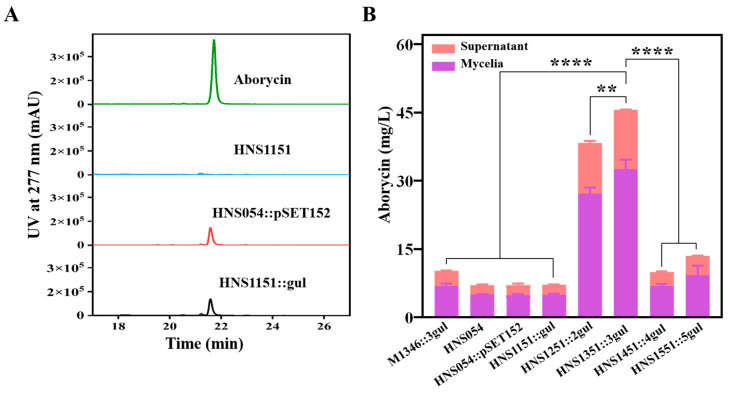
Improvement of aborycin production. (**A**) Comparative HPLC analysis of aborycin in the culture extracts of corresponding strains. (**B**) Titers of different strains. ****: *p* < 0.0001; **: *p* < 0.01.

The fermentation broth of engineered strains was compared for aborycin production (Figure 3A). Both supernatant and mycelial analyses demonstrated that strains HNS054 and HNS054::pSET152 produced approximately 7.1 ± 0.1 mg/L of aborycin (Figure 3B). In contrast, strains with two or three copies of the aborycin BGC produced 38.4 ± 0.9 mg/L (HNS1251::2gul) or 45.6 ± 2.1 mg/L (HNS1351::3gul), respectively. Interestingly, strains with four or five copies of the aborycin BGC did not exhibit further production enhancement; instead, a decrease was observed. This suggested that the overexpression of the aborycin BGC genes was no longer the limiting factor for aborycin production. To achieve further improvements, additional metabolic engineering strategies, such as enhancing precursor availability and antibiotic tolerance, need to be considered.

The enhancement of aborycin production in strain HNS1351::3gul was significant, representing a 6.4-fold increase compared to native HNS054. In comparison, the three-copy integration counterpart in *S. coelicolor*, strain M1346::3gul, produced aborycin at 10.2 ± 0.7 mg/L. Consequently, the aborycin yield of strain HNS1351::3gul surpassed that of strain M1346::3gul by 4.5-fold. These results highlight the effectiveness of the MSGE strategy in HNS054-based hosts, leading to a remarkable improvement in aborycin production.

### 2.8. Improvement of Actinorhodin Production

**Figure 4:**
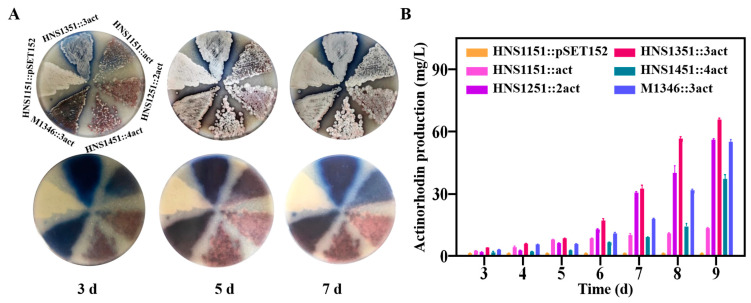
Detection and comparison of actinorhodin concentrations in strains. (**A**) Visual observation of actinorhodin production. (**B**) Quantification of actinorhodin concentrations.

The actinorhodin BGC was incorporated into the chromosomes of strains HNS1151-1551 using the plasmid pSET152::act, generating strains HNS1151::act, HNS1251::2act, HNS1351::3act, and HNS1451::4act, respectively (Figure S16). Unfortunately, strain HNS1551::5act was not obtained, possibly due to limitations in integration capacity (Figure S17). The actinomycin yield gradually increased with the extension of culture time (Figure 4). Strain HNS1351::3act, which harbored three copies of the actinorhodin BGC, exhibited more intense pigmentation on MS agar media relative to other strains HNS054-based hosts and M1346::3act, indicating higher actinorhodin production (Figure 4A). On day 9, strain HNS1351::3act produced actinorhodin at a 4.9-fold, 1.2-fold, and 1.2-fold higher rate than strains HNS1151::act, HNS1251::2act, and M1346::3act, respectively (Figure 4B). However, actinorhodin production decreased by 43% in strain HNS1451::4act, which had four copies of the actinorhodin BGC. The expression of the empty plasmid pSET152 in strain HNS1151 had no effect on actinorhodin production. These results highlight the superiority of HNS054-based hosts in heterogeneously expressing foreign BGCs.

## 3. Discussion

The genomic sequencing of HNS054 has revealed intricate insights into its biological information, revealing a clear genome structure and rich functional potential. HNS054 harbors 6678 putative protein-coding genes, showcasing a relatively high gene density. Functional annotation using COG, GO, and KEGG databases classified the genes into various categories and pathways, reflecting the diverse metabolic capabilities of this strain. The genome size of HNS054 is 7.5 Mb, falling within the typical range of conventional *Streptomyces* genomes (6–12 Mb). Notably, it is comparable to the genome sizes of well-known *Streptomyces* chassis strains, such as *S. coelicolor* A3(2) (8.7 Mb), *S. griseus* NBRC 13350 (8.5 Mb), *S. albus* J1074 (6.8 Mb), and *S. atratus* SCSIO ZH16 (9.6 Mb) [21,22]. In comparison, the smallest reported *Streptomyces* genome is that of *S. xiamenensis* 318, with a size of 5.96 Mb and 21 BGCs [23]. The relatively moderate genome size of HNS054 positions it favorably for genome-scale modeling and engineering, facilitating the optimization of its metabolic performance and productivity. Furthermore, HNS054 exhibits fewer BGCs compared to the aforementioned chassis strains, which have 30, 38, 22, and 26 BGCs, respectively. This suggests that HNS054 boasts a cleaner metabolic background, reducing interference and competition from endogenous secondary metabolites with heterologous ones.

Despite exhibiting rapid growth compared to *S. coelicolor* M1146, unraveling the underlying mechanisms through genome comparison alone proves challenging. Previous reports suggest a positive correlation between rRNA operon and tRNA gene count with bacterial growth rate and metabolic activity [24]. While HNS054 possesses more ribosomal operons (6) and tRNA genes (72) than *S. xiamenensis* 318 (5 and 55, respectively), these values fall within the range observed in common chassis strains (6–7 rRNA operons, 63–69 tRNA genes). HNS054 also shares a characteristic symmetrical distribution of conserved genes around the OriC with these chassis strains. This arrangement potentially facilitates early expression of essential genes and discourages subtelomeric rearrangements, enhancing strain stability and adaptability [21,22,23]. Elucidating the precise genomic basis of HNS054’s rapid growth requires the application of contemporary functional genomics approaches.

It has been reported that salt-resistant *Halomonas* spp. are preferred hosts due to their ability to conduct contamination-free fermentation without the need for strict sterilization under high salt conditions [25]. While HNS054 may not exhibit resistance levels as high as *Halomonas* spp. (moderate strains can tolerate 30–150‰ NaCl (*w*/*v*), and extreme strains can tolerate >200‰ NaCl (*w*/*v*)), HNS054 demonstrated faster growth and higher bioaccumulation than *S. coelicolor* M1146 in the salinity range of 0–45‰ NaCl (*w*/*v*) (Figure 2). Furthermore, HNS054-derived strains show a lower susceptibility to contamination than M1146-derived strains during fermentation, genetic engineering, and sporing under 35‰ salinity. It can be deduced that the large-scale fermentation performance of the engineered HNS054 would reduce the risk of bacterial contamination under optimized salinity conditions.

HNS054 shines not only in growth and salt tolerance but also in its inherent potential for genetic manipulation. Compared to other *Streptomyces* chassis, its enriched repertoire of natural *attB* sites synergizes with its conventional SSR system (Table S5) and facilitates advanced multiplex site-specific genome editing [20]. Furthermore, improved HNS054-derived strains were constructed via CRISPR/Cas9-mediated precise deletion of specific BGCs and insertion of artificial φC31 *attB* sites. Notably, removing *S. griseoincarnatus* HNS054’s native secondary metabolite BGCs and introducing *attB* sites maintained its growth and morphology (Figure S12), indicating a simplified background, potentially leading to increased precursor availability and secondary metabolite production. The presence of multiple homologous *attB* sites enables these HNS054-based hosts to leverage the “one integrase-multiple *attB* sites” concept for the MSGE strategy. This approach has proven to enhance secondary metabolite production in various *Streptomyces* strains [5,7,15,26,27]. Thus, HNS054’s genomic features and amenability to MSGE make it a promising platform for overproducing valuable secondary metabolites.

However, the results indicate that the MSGE method has limitations in two key aspects. Firstly, as the number of *attB* sites of the same type increased from one to five, the conjugation efficiency of HNS054-based hosts and *S. coelicolor* M1146-based hosts declined from thousands to single conjugon (Figure S17). This observation suggests that introducing foreign gene clusters using the MSGE method becomes more challenging with an increasing number of *attB* sites of the same type in a strain. Secondly, in both case studies, an increase in the BGC copy number led to a declining trend in yield (Figure 3B and Figure 4B), a phenomenon consistent with similar reports. For instance, strain *S. albus* B4, which harbors four copies of pyridinopyrone A, aloesaponarin II, and didemethoxyaranciamycinone BGCs, respectively, exhibited lower production than strain *S. albus* B2P1, which has three copies of the corresponding BGCs [7]. The mechanism behind this phenomenon is unclear, but it may involve the strain’s self-protection mechanism. These limitations underscore the necessity of employing additional metabolic engineering strategies to overcome diminishing returns associated with higher BGC copy numbers.

While overexpression of the aborycin BGC genes in HNS1451 did not lead to high aborycin production, we suspect that the supply of amino acids, especially noncanonical amino acids, might be a limiting factor. This aligns with the study by Schulz et al. [28], who demonstrated a significant increase in FK506 production by optimizing pipecolate supply in *S. tsukubaensis*. Similarly, optimizing the noncanonical amino acid synthesis pathway in HNS1451 could potentially unlock further improvements in aborycin production.

Compared to the well-established model strain, *S. coelicolor* M145, and its derived strains, marine actinomycetes possess a unique metabolic system that facilitates the discovery and development of novel drug lead compounds from the marine environment. Zhang et al. [9] eliminated salinosporamide synthesis in the marine strain *S. tropica* CNB-440 and introduced the phage φC31 *attB* site of *S. coelicolor*, generating strain *S. tropica* CNB-4401. The thiolactinomycin BGC from *S. pacifica* was heterologously expressed, and the thiolactomycin production in *S. tropica* CNB-4401 was approximately three-fold that of *S. coelicolor* M1152. Yang et al. [10] disrupted the synthetic pathway of two main metabolites (ilamycins and atratumycin) in *S. atratus* SCSIO ZH16 and used the natural specific integration site of this strain, resulting in the marine *Streptomyces* expression platform MGCEP 1.0. Alkaloids, aminonucleosides, non-ribosomal peptides, and polyketides BGCs were heterologously expressed in MGCEP 1.0, leading to the identification of 19 compounds.

Aborycin, a novel anti-HIV metabolite with activity against various Gram-positive bacteria, holds promise for treating infectious diseases [15,29]. Its unique structure makes it a valuable candidate for drug development. However, its natural production is limited. In this study, we successfully increased aborycin production in a marine *Streptomyces* strain using the MSGE strategy. This significant increase (6.4-fold compared to the native strain) could facilitate further investigation and optimization of aborycin’s bioactivity and pave the way for its potential development as a therapeutic agent.

Being a marine actinomycete, HNS054 boasts faster growth and high salinity tolerance. The manipulation of HNS054 and its derivative strains offers notable advantages, including ease of operation, reduced susceptibility to contamination, and expedited completion of conjugation experiments. These inherent traits of the HNS054 series not only facilitate genetic engineering manipulations but also position them as promising candidates for future large-scale fermentation processes.

## 4. Materials and Methods

### 4.1. Strains, Plasmids, Primers, and Culture Conditions

The primers, plasmids, and strains utilized in this study are outlined in Tables S7 and S8. Yeast extract and tryptone were purchased from Oxoid (Cambridge, UK). Mannitol, sucrose, glucose, NaOH and KNO_3_ were purchased from Sinopharm Chemical Reagent Co., Ltd. (Shanghai, China). Peptone, K_2_HPO_4_, agar and other reagents was purchased from Sangon Biotech Co., Ltd. (Shanghai, China). *Streptomyces* were cultured either on mannitol soya flour agar medium (MS, 20 g/L mannitol, 20 g/L soy flour, 15 g/L sea salt, 20 g/L agar) or in R5 liquid medium (10 g/L glucose, 0.1 g/L casamino acids, 5 g/L yeast extract, 5.73 g/L [Tris(hydroxymethyl)methyl]-2-aminopropanesulfonic acid (TES), 10 mL of 0.5% KH_2_PO_4_, 4 mL of 5 M CaCl_2_.2H_2_O, 15 mL of 20% L-proline, and 7 mL of 1 M NaOH, 2 mL trace element solution) with suitable antibiotics. *Escherichia coli* were cultured at 37 °C in Luria–Bertani (LB) medium supplemented with appropriate antibiotics with shaking at 300 rpm for 16 h. *Streptomyces* was cultivated on MS salt agar at 28 °C for 7 d for sporulation. Liquid cultures were involved with ISP2 medium (10 g/L yeast extract, 10 g/L malt extract, 4 g/L glucose, 30 g/L sea salt), YEME medium (3 g/L yeast extract, 5 g/L peptone, 3 g/L malt extract, 10 g/L glucose, 340 g/L sucrose, 30 g/L sea salt), Gauze’s Medium No.1 (1 g/L KNO_3_, 0.5 g/L K_2_HPO_4_, 0.5 g/L MgSO_4_·7H_2_O, 0–100 g/L NaCl, 0.01 g/L FeSO_4_·7H_2_O, 20 g/L soluble starch) or R5 medium and incubated at 28 °C with shaking at 200 rpm for 5–7 d.

### 4.2. Genome Sequencing, Assembly and Annotation

The genome sequencing of *Streptomyces* sp. HNS054 was conducted by Biomarker Technologies (Beijing, China) using Pacbio Sequel II and Illumina NovaSeq 6000 platform. The assembly of long reads was executed using Hifiasm v0.12 [30], resulting in a single contig spanning 7.5 Mb. Quality control for short reads was conducted through Fastuniq V1.1 (https://sourceforge.net/projects/fastuniq/, accessed on 22 September 2022), followed by trimming with Adapter Removal [31]. To rectify errors in long reads, SOAPdenovo2 v2 (https://github.com/aquaskyline/SOAPdenovo2, accessed on 22 September 2022) with a k-mer setting of 17 was applied to the trimmed reads. The corrected long reads underwent further refinement with Pilon v1.22 (https://github.com/broadinstitute/pilon, accessed on 22 September 2022) and circularization with Circlator v1.5.5 (https://github.com/sanger-pathogens/circlator, accessed on 23 September 2022), culminating in the acquisition of the complete genome sequence of HNS054. The complete genome sequence has been submitted to the NCBI GenBank database under the accession number CP139576.1. The 16S rRNA gene sequence has also been submitted to the NCBI Nucleotide database under the accession number OR898379.1.

Coding genes were identified using Prodigal v2.6.3 (https://github.com/hyattpd/Prodigal, accessed on 23 September 2022). Infernal v1.1.3 (http://eddylab.org/infernal/, accessed on 26 September 2022) was employed for the prediction of rRNA genes, while tRNAscan-SE v2.0 (https://github.com/UCSC-LoweLab/tRNAscan-SE, accessed on 26 September 2022) predicted tRNA genes. Additionally, Infernal v1.1.3 was utilized for the prediction of other non-coding RNAs. CRISPRs and Cas genes were discerned using CRISPRCasFinder (https://crisprcas.i2bc.paris-saclay.fr/CrisprCasFinder/Index, accessed on 12 October 2022). For functional annotation, the predicted protein underwent a blast analysis against diverse databases, including Nr (Non-Redundant Protein Sequence Database), Swiss-Prot, Pfam, TrEMBL (Translation of EMBL), KEGG (Kyoto Encyclopedia of Genes and Genomes), and eggNOG (evolutionary genealogy of genes: Non-supervised Orthologous Groups). GO (Gene Ontology) annotation was carried out using Blast2go v2.5 (https://www.blast2go.com/, accessed on 10 October 2022). Orthologous proteins were predicted and clustered across the genomes using the OrthoVenn2 (http://www.bioinfogenome.net/OrthoVenn/start.php, accessed on 13 October 2022), employing the OrthoMCL algorithm. AntiSMASH 7.1.0 (https://antismash.secondarymetabolites.org/, accessed on 17 October 2022) was employed for predicting secondary metabolite BGCs and forecasting the synthesis of metabolic products in HNS054.

RepeatMasker v4.0.5 (https://www.repeatmasker.org/RepeatMasker/, accessed on 13 October 2022) was employed for predicting repetitive sequences. IslandViewer 4 (http://www.pathogenomics.sfu.ca/islandviewer/, accessed on 24 November 2022) was used to identify GIs. The OriC was determined through Ori-Finder 2022 (http://tubic.tju.edu.cn/Ori-Finder/, accessed on 24 November 2022). SSR system *attB* loci were identified by employing local blast to search for the core regions of nine prevalent types of SSR system *attB* nucleotide sequences within the HNS054 genome.

### 4.3. Comparative Genomic Analysis

The Mauve 2.3.1 (http://darlinglab.org/mauve/mauve.html, accessed on 15 November 2022) was utilized for the creation and visualization of multiple genome alignments. The phylogenetic tree based on the 16S rRNA gene was constructed using a neighbor-joining approach in MEGA 11, with 1000 bootstrap replicates. dDDH estimates were determined using the Genome-to-Genome Distance Calculator (GGDC) web server (https://ggdc.dsmz.de/, accessed on 20 November 2022). ANI values were calculated by the JSpeciesWS online service (https://jspecies.ribohost.com/jspeciesws/#analyse, accessed on 20 November 2022). To conduct pan-genome analysis for strain HNS054 and other *Streptomyces* species, the Bacterial Pan Genome Analysis Pipeline (BPGA v1.3, http://www.iicb.res.in/bpga/index.html, last accessed on 10 December 2023) software was employed and constructed a whole-genome-based phylogenetic tree. The resulting phylogenetic tree was visualized using the online software iTOL (Interaction Tree of Life, https://itol.embl.de/, last accessed on 10 December 2023). Detailed information regarding *Streptomyces* species utilized for comparative genomic analysis, along with their GenBank accession numbers, is available in Table S9.

### 4.4. Assessment of Growth, Salt Tolerance, and Antibiotic Sensitivity

The biomass of HNS054 and *S. coelicolor* M1146 in the fermentation broth was determined utilizing a simplified diphenylamine colorimetric method [32].

Salt tolerance testing of the strains involved the preparation of Gauze’s Medium No.1 with varying NaCl concentrations (0‰, 15‰, 30‰, 45‰, 60‰, 80‰, and 100‰). The tested strains were then inoculated onto the respective media and cultured at 28 °C for 5–7 d. The growth of the strains was monitored, and the results were documented.

The antibiotic sensitivity of HNS054 was tested on MS salt agar plates with various concentrations of antibiotics. Kanamycin, ampicillin, chloramphenicol, apramycin, nalidixic acid, thiostrepton, and tetracycline were assessed with concentrations ranging from 0–100 μg/mL. Additionally, hygromycin and spectinomycin were examined with concentrations ranging from 50–300 μg/mL. The spore suspension of HNS054 was inoculated on the plates at 28 °C for 5–9 d. The growth of the strain on the plates was observed to determine its antibiotic sensitivity.

### 4.5. CRISPR/Cas9 and Construction of the HNS054-Derived Hosts

CRISPR/Cas9 technology offers several advantages for *Streptomyces* genome editing, including higher efficiency, easier operation, and faster turnaround times. This technology has significantly advanced genetic engineering in *Streptomyces* [33]. The construction of HNS054-derived hosts involved the CRISPR/Cas9 genome editing method to generate a series of strains (HNS1151-HNS1551). The strain HNS1151 was generated by knocking out BGC11 to eliminate aborycin production. Subsequently, in strains HNS1251-HNS1551, artificial φC31 *attB* sites were incorporated at the locations of deleted target BGCs (e.g., BGC11, BGC14, BGC17, BGC2). The general construction process is outlined below:

1. Amplification of upstream and downstream regions: PCR amplification of the upstream and downstream regions of the targeted BGCs (e.g., BGC11, BGC14, BGC17, BGC2) using specific primer pairs (e.g., Del-BGC11-up-B-fwd/rev, Del-BGC11-down-B-fwd/rev).
2. Amplification of sgRNA expression cassette: PCR amplification of the sgRNA expression cassette using a plasmid template (e.g., pKCcas9dO) and specific primers (e.g., BGC11-sgRNA-B-fwd, sgRNA-rev).
3. Gibson assembly: assembly of the three DNA fragments (upstream region, downstream region, sgRNA expression cassette) using the Gibson assembly method to generate plasmids (e.g., pKY01dB11::attB).
4. Plasmid introduction: introduction of the constructed plasmids into the HNS054-derived hosts through conjugal transfer on MS solid media.
5. Verification of double crossovers: verification of correct double crossovers in the transformed strains by PCR using specific primers (e.g., ID-BGC11-B-fwd/rev).

This general strategy was repeated to construct strains HNS1251, HNS1351, HNS1451, and HNS1551, each with an increasing number of artificial φC31 *attB* sites in the locations of the deleted BGCs. The resulting strains serve as engineered hosts with modified genomic features for further studies or applications.

### 4.6. Production of Aborycin

The plasmid pSET152::gul, harboring the aborycin BGC, was constructed using a one-step cloning method. Firstly, the pSET152 plasmid was linearized by PCR using the primers pSET152-fwd/pSET152-rev. Subsequently, the aborycin BGC was amplified from the genome DNA of *S. griseoincarnatus* HNS054 using the primers 054 gul-fwd/054 gul-rev. Finally, the aborycin BGC and the linearized pSET152 were assembled into the circular plasmid pSET152::gul using a one-step cloning kit.

The plasmid pSET152::gul was transferred into strains HNS1151-1551 by intergeneric conjugation with *E. coli* ET12567/pUZ8002 as the donor. Exconjugants were acquired by selecting for resistance to apramycin and were subsequently verified through PCR analysis using the primers ID-oriT-fwd/Native B-rev, ID-oriT-fwd/BGC11 B-rev, ID-oriT-fwd/BGC14 B-rev, ID-oriT-fwd/BGC17 B-rev, and ID-BGC2-fwd/BGC2 B-rev to confirm *attP*-*attB* recombination, respectively. In parallel, the plasmid pSET152 was introduced into strain HNS054 as a control.

Aborycin production in the engineered strains and *S. coelicolor* M1346::3gul were assessed using the same methodologies [15].

The *Streptomyces* sp. strains were inoculated into YEME medium containing appropriate antibiotics and incubated at 200 rpm, 28 °C for 6–9 d until fermentation was complete. The fermented broth was centrifuged to separate the supernatant from the mycelia, and then each fraction was extracted separately. The supernatant was adsorbed by macroporous resin AB-8. The mycelium was extracted with methanol and then concentrated by rotary evaporation. The extracts from the supernatant and the mycelia were filtered through 0.22 µm membranes before HPLC or LC-MS/MS analysis. Analytical HPLC was performed on a Shimadzu Prominence LC-20A system (Shimadzu, Tokyo, Japan) equipped with a Ultimate XB-C18 column (4.6 × 250 mm, 5 μm, 300Å Welch, Shanghai, China). The elution was carried out at a flow rate of 1 mL/min and monitored at 277 nm, using 1% solvent B (0.1% formic acid in acetonitrile) for 0–7 min, followed by a linear gradient to 95% solvent B for 7–30 min (solvent A: 0.1% formic acid in H_2_O).

The positive ion peak of aborycin in the mass spectra was detected by UPLC-MS/MS on a Dionex U3000 RSLC system (Thermo Scientific, Waltham, MA, USA) coupled to a Q Exactive Spectrometer. The separation was performed on a Ultimate XB-C18 column (4.6 × 250 mm, 5 μm, Welch, Shanghai, China) at a flow rate of 1 mL/min, using the following gradient elution: 5% solvent B (0.1% formic acid in acetonitrile) for 0–5 min; 5–30% solvent B for 3–8 min; 30–60% solvent B for 8–25 min; 60–100% solvent B for 25–30 min and 100% solvent B for 5 min (solvent A: 0.1% formic acid in H_2_O).

Strain M1346::3gul was cultured in R5 medium at 200 rpm and 28 °C for 9 d to complete the fermentation. The supernatant was enriched by AB-8 resin and then purified by semipreparative HPLC using a YMC-PACK ODS-A column (10 × 250 mm, 5 µm, YMC, Kyoto, Japan). Fractions F1–F4 near the target position were collected and concentrated by rotary evaporation. Fraction F2 was further analyzed. Fraction F2 was dissolved in 2 mL of methanol and subjected to analytical HPLC. The target peaks were collected and lyophilized to obtain pure aborycin as a white powder. Aborycin was dissolved in methanol to a concentration of 1 mg/mL and serially diluted. The dilutions were analyzed by HPLC and a standard curve of aborycin concentrations versus peak areas was constructed.

### 4.7. Production of Actinorhodin

The plasmid pSET152::act, harboring the actinorhodin BGC [34], was introduced into strains HNS1151-1551 through conjugal transfer. Exconjugants were obtained by selecting for apramycin resistance and confirmed via PCR analysis using the aforementioned primer pairs to verify *attP*-*attB* recombination. Plasmid pSET152 was also transferred into strain HNS1151 as a control. To assess whether the actinorhodin production ability of HNS054-based hosts is comparable to that of *S. coelicolor* M1346, the plasmid pSET152::act was additionally transferred into *S. coelicolor* M1346, generating the strain *S. coelicolor* M1346::3act.

The quantification of actinorhodin production in the engineered strains and *S. coelicolor* M1346::3act was conducted using the following method. The presence of the antibiotic was indicated by the blue coloration of actinorhodin on MS agar media. The strains were inoculated into YEME medium containing appropriate antibiotics and incubated at 200 rpm, 28 °C for 9 d. For actinorhodin extraction, 1 mL of culture samples was mixed with 250 μL of 5 M KOH and then centrifuged at 12000 rpm for 5 min. The concentration of actinorhodin in the supernatant was determined by measuring the absorbance at 640 nm, employing an extinction coefficient of 2.53 × 10^4^ L/(mol·cm) [35].

### 4.8. Statistical Analysis

All of the results were expressed as the mean ± standard deviation (SD), which resulted from at least three independent experiments. The statistical analysis was performed using one-way ANOVA. The significance level was set at *p* < 0.05. The software used for the analysis was GraphPad Prism 8.3.

## 5. Conclusions

This study presents the comprehensive genomic analysis and annotation of the marine actinomycete *S. griseoincarnatus* HNS054. The genome of HNS054 revealed its phylogenetic position, diverse repertoire of secondary metabolites, and multiple site-specific recombination system *attB* sites. The strain exhibits high salinity tolerance, a fast growth rate, high biomass production, and sensitivity to antibiotics, which are desirable traits for industrial applications. Furthermore, a series of HNS054-based hosts were constructed by deleting BGCs and introducing the φC31 *attB* sites. Amplification of the aborycin and the actinorhodin BGCs in engineered strains significantly improved the production of these antibiotics. This study identifies HNS054 as a promising foundation for further developing industrial chassis adept at overproducing marine natural products.

## Data Availability

The original data are included in the paper and can be obtained from the corresponding author upon reasonable request.

## Supplementary Materials

The supporting information can be downloaded at: https://www.mdpi.com/article/10.3390/ijms25063127/s1. References [36,37,38,39] are cited in Supplementary Materials.
