# Supplementary material for: Genomic Insights and Synthetic Biology Applications of Marine Actinomycete Streptomyces griseoincarnatus HNS054
Source: Int J Mol Sci. 2024 Mar 8;25(6):3127. doi: 10.3390/ijms25063127 (PMC10970339; doi:10.3390/ijms25063127)
Supplement: Supplementary file 1 [file ijms-25-03127-s001.zip › ijms-2853665-supplementary.pdf]

# Supporting Materials

## Genomic Insights and Synthetic Biology Applications of Marine Actinomycete *Streptomyces griseoincarnatus* HNS054

**Qinghua Wang [1,2], Jing Zhao [1,2,3], Zhaoyuan Liu [1,2], Shaoxiong Ding [1,2,3], Zhiyong Huang [4] and Jun Chen [1,2,3,*]**

[1] State Key Laboratory of Marine Environmental Science, Xiamen University, Xiamen 361102, China; wangqinghua@stu.xmu.edu.cn (Q.W.); sunnyzhaoj@xmu.edu.cn (J.Z.); liuzhaoyuan2919@163.com (Z.L.); sxding@xmu.edu.cn (S.D.)
[2] Department of Marine Biological Science and Technology, College of Ocean and Earth Sciences, Xiamen University, Xiamen 361102, China
[3] State-Province Joint Engineering Laboratory of Marine Bioproducts and Technology, Xiamen University, Xiamen 361102, China
[4] Tianjin Institute of Industrial Biotechnology, Chinese Academy of Sciences, Tianjin 300308, China; huang_zy@tib.cas.cn
* Correspondence: chenjun@xmu.edu.cn

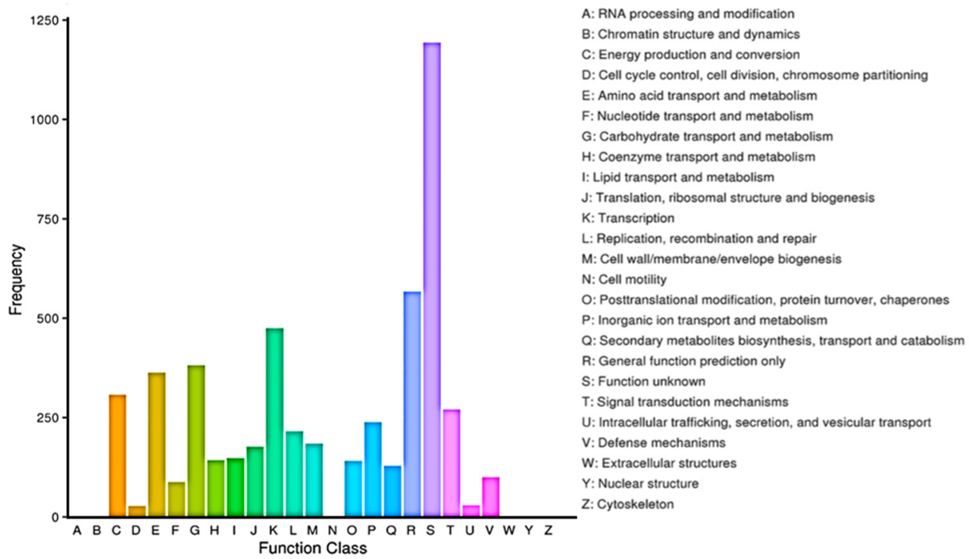


**Figure S1.** COG annotation of the HNS054 genome. Functional categories were assigned to 5121 genes in the HNS054 genome using the COG annotation. The majority of these genes fell into categories such as S (function unknown), R (general function prediction only), K (transcription), G (carbohydrate transport and metabolism), E (amino acid transport and metabolism), C (energy production and conversion), and T (signal transduction mechanisms). Notably, 129 genes were associated with secondary metabolite biosynthesis, transport, and metabolism (Q).

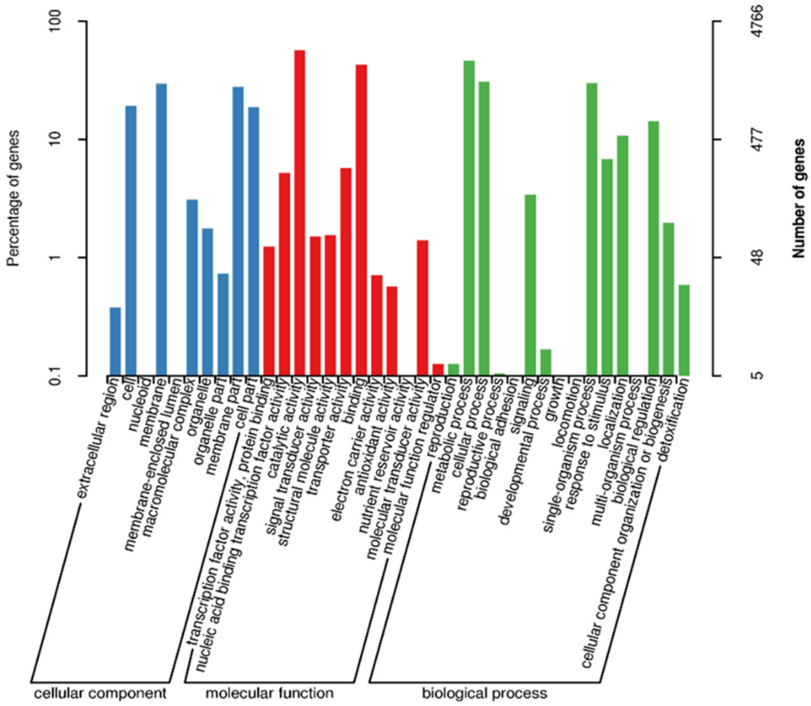


**Figure S2.** GO annotation of the HNS054 genome. Using the GO database annotation, 4766 genes were categorized into three major groups: cellular component, molecular function, and biological process. Enriched gene categories included membrane part, cell, membrane, and macromolecular complex (cellular component), as well as catalytic activity and binding (molecular function). Additionally, genes were associated with metabolic process, single-organism process, and biological process (biological process).

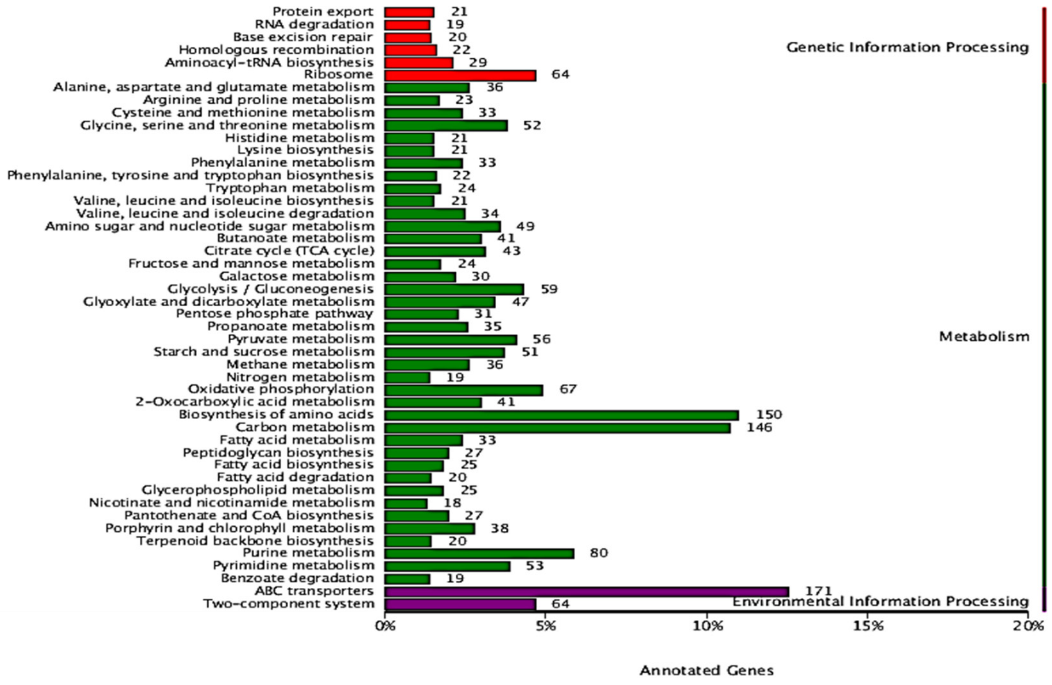


**Figure S3.** KEGG annotation of the HNS054 genome. The KEGG database annotated 2380 genes, classifying them into three main categories: genetic information processing, metabolism, and environmental information processing. The predominant genes were identified in ABC transporters, amino acid biosynthesis, and carbon metabolism, with notable contributions from purine metabolism and two-component systems.

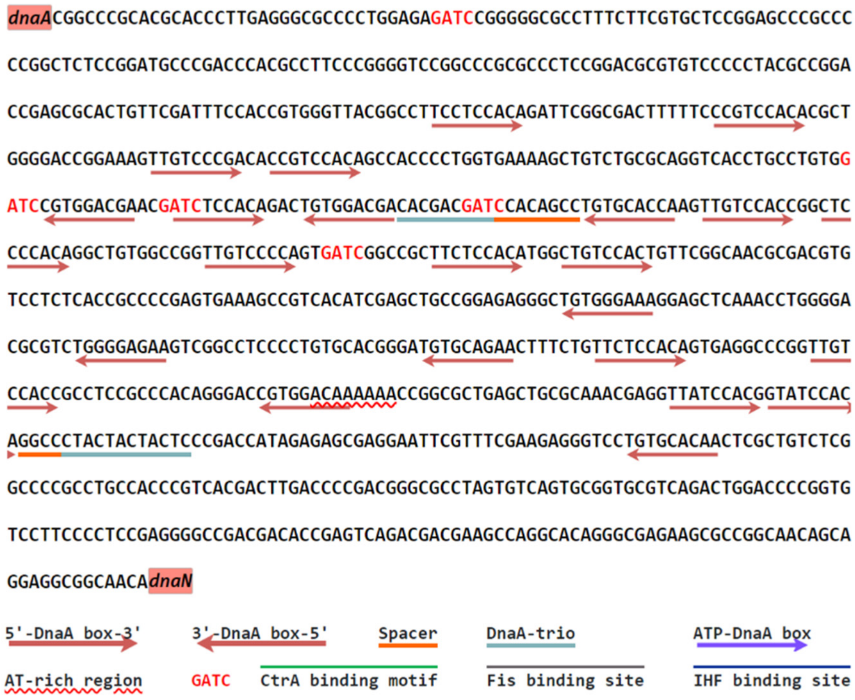


**Figure S4.** Characterization of the replication origin site (OriC) on the HNS054 chromosome. The replication origin comprises an AT-rich region (ACAAAAAA), 22 DnaA boxes, five GATC sites, and two DnaA-trios.

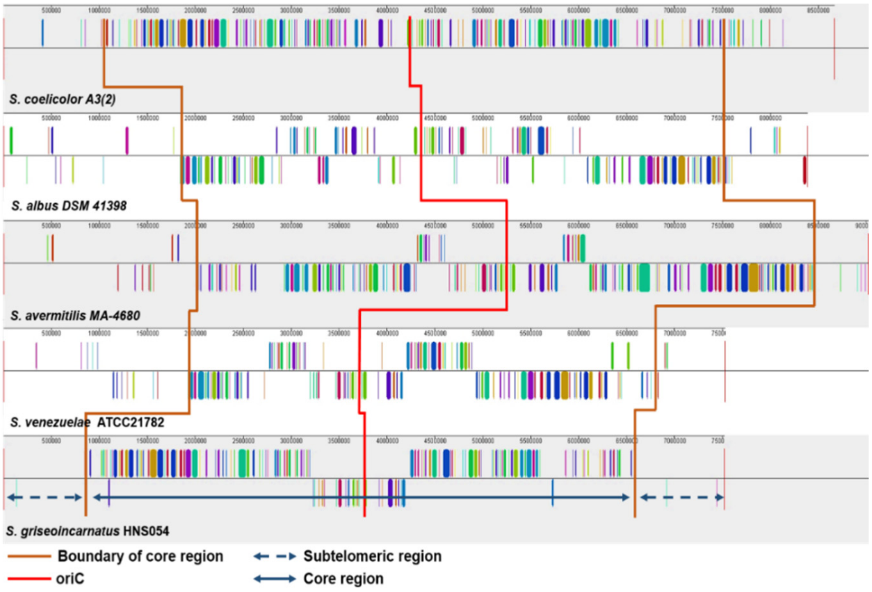


**Figure S5**. Multigenome comparison of HNS054 and other *Streptomyces* chromosomes.

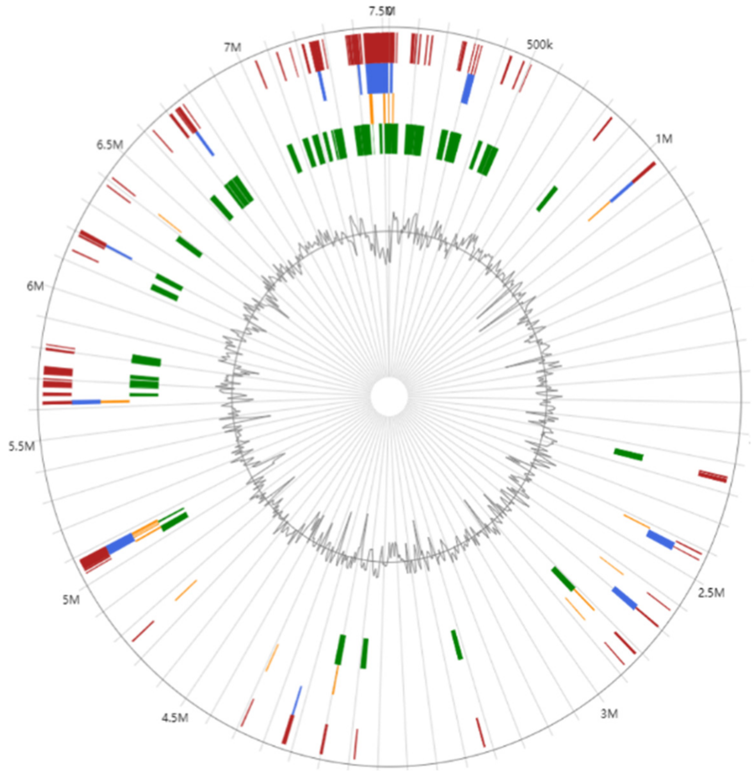


**Figure S6.** Genomic island (GI) analysis of the HNS054 genome. The core region exhibits highly conserved and homologous genes, while the accessory genome includes dispensable elements, including 18 GIs predicted by IslandViewer4. The red module corresponds to comprehensive analysis, the green module to IslandPick analysis, the yellow module to SIGI-HMM analysis, and the blue module to IslandPath-DIMOB analysis.

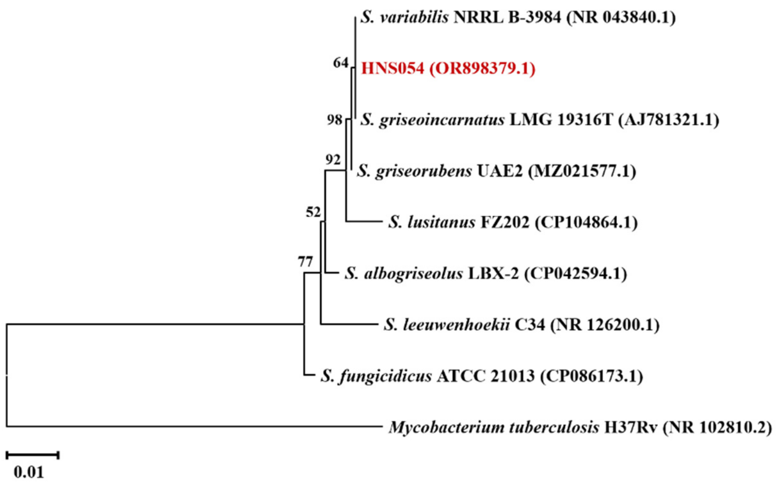


**Figure S7.** Neighbor-joining phylogenetic tree based on 16S rRNA gene sequences of HNS054. In 2021, following the reclassification of the *Streptomyces* genus (https://lpsn.dsmz.de/genus/streptomyces), *S. variabilis* was officially recognized as a heterotypic synonym of *S. griseoincarnatus*.

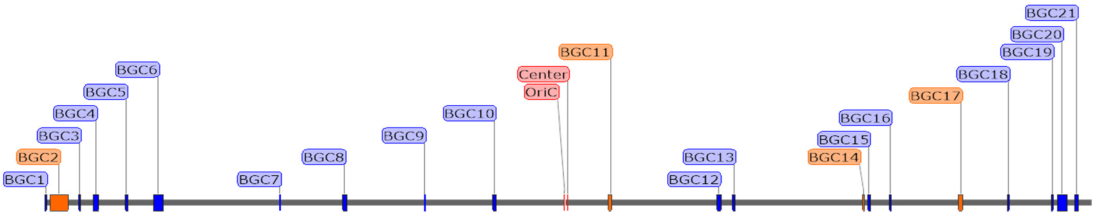


**Figure S8.** Distribution of BGCs in the HNS054 genome. The antiSMASH web server was employed to upload the HNS054 genome sequences and identify individual BGCs, followed by a comprehensive examination of their genomic locations. A total of 21 BGCs were found to be evenly distributed across the genome.

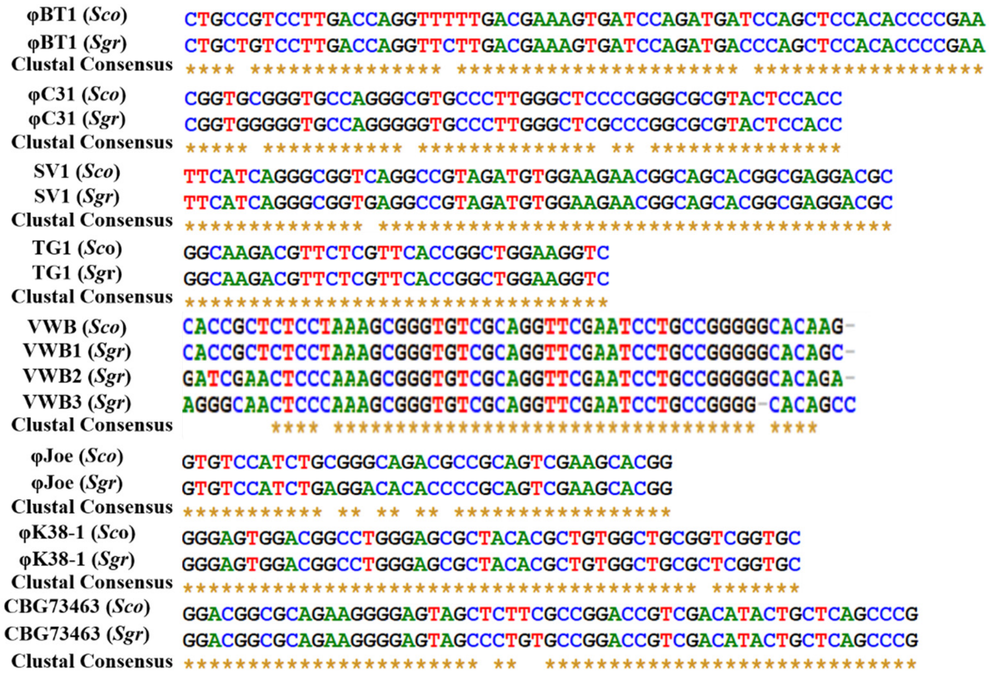


**Figure S9.** Nucleotide sequence alignment at *attB* sites of HNS054. *Sco*: *S. coelicolor*; *Sgr*: *S. griseoincarnatus*.

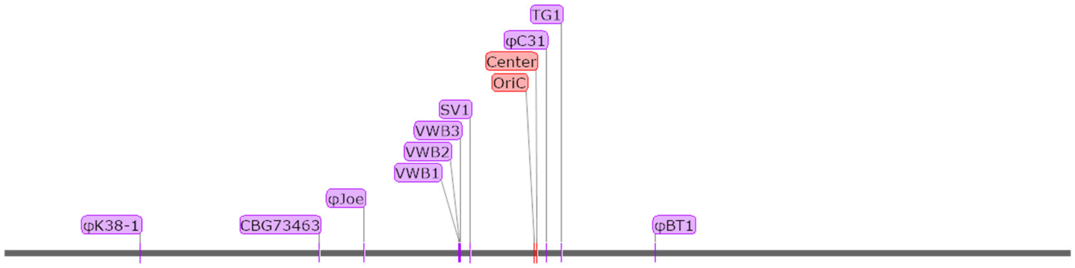


**Figure S10.** Distribution of 10 natural *attB* sites in the HNS054 chromosome.

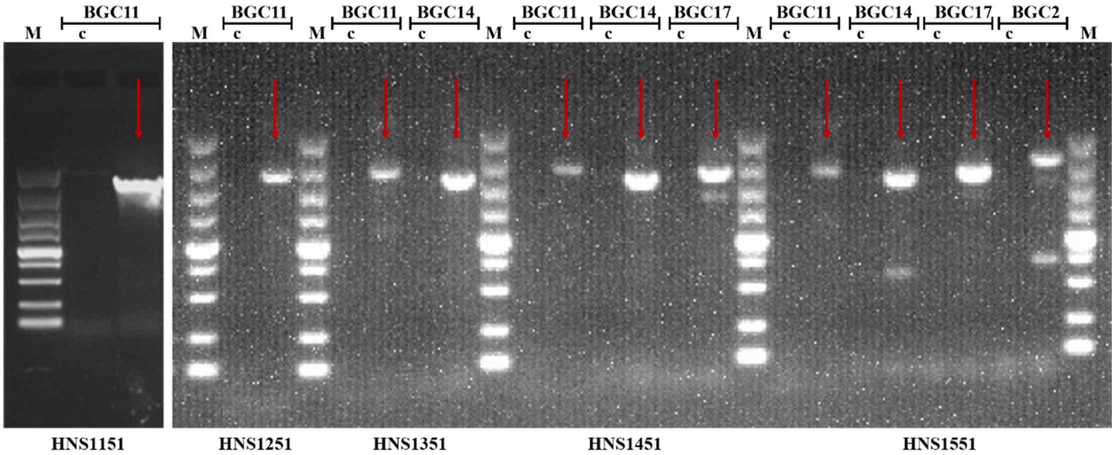


**Figure S11.** PCR verification of strains featuring BGC knockout and φC31 *attB* site introduction. M: 5 Kb DNA marker; c: negative control.

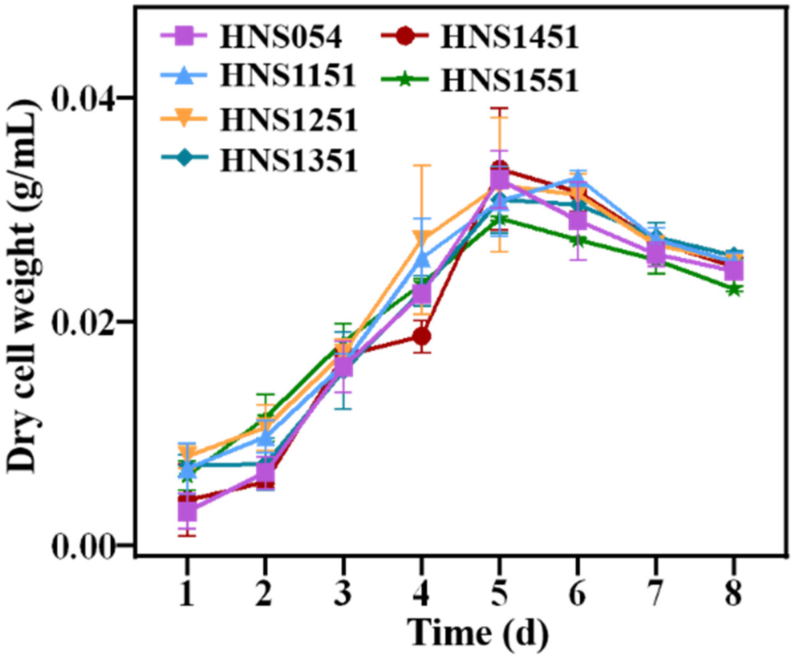


**Figure S12.** Growth curve of strains. *Streptomyces* spores were scraped from MS plates and inoculated into the seed medium for 24 h. The seed culture was then transferred to the fermentation medium to achieve an initial $OD_{600}$ of 0.2. 1 mL samples were collected every 24 h and centrifuged. The supernatant was discarded and the pellets were dried at 65 °C for 3-5 d until the weight was constant. The biomass weight at different time points was calculated and the growth curve was plotted.

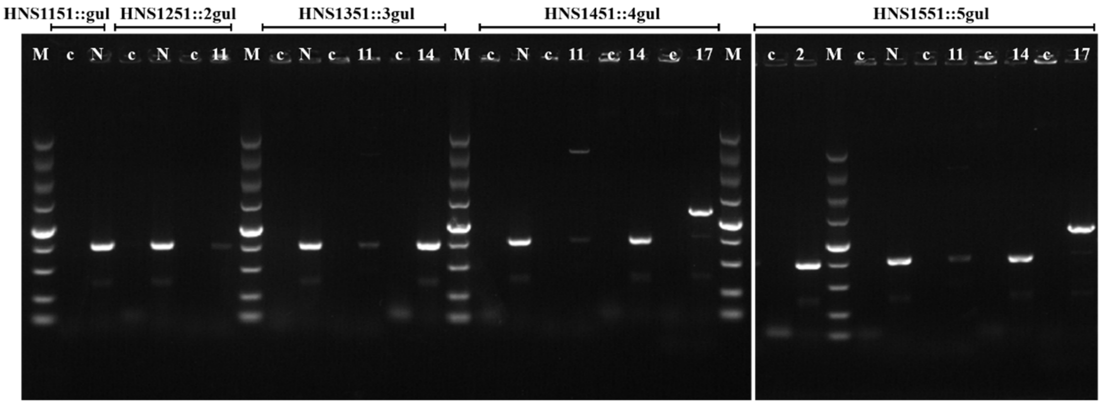


**Figure S13.** Identification of the plasmid pSET152::gul introduced into the strains HNS1151-1551. M: 5 Kb DNA marker; c: negative control. N: native; 11: BGC11; 14: BGC14; 17: BGC17; 2: BGC2.

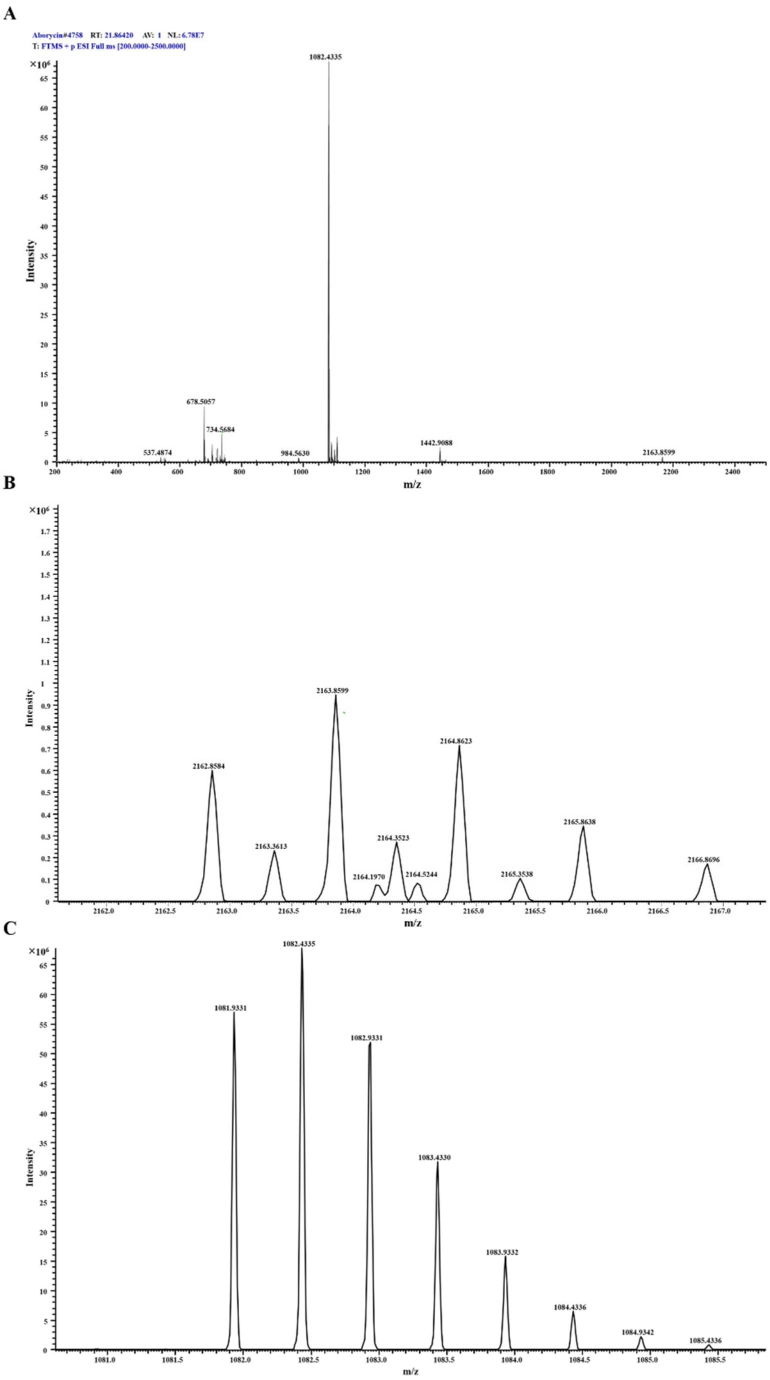


**Figure S14.** Positive ion peak in the mass spectra of aborycin. The ESI-MSMS data revealed [M + H]$^+$ at m/z = 2162.8584, calculated for C97H132N23O26S4: 2162.8591, and [M + H]$^{2+}$ at m/z = 1082.4335. Figure S14A displays the full mass spectrum at 21.9 min, while Figure S14B-C present magnified views of Figure S14A.

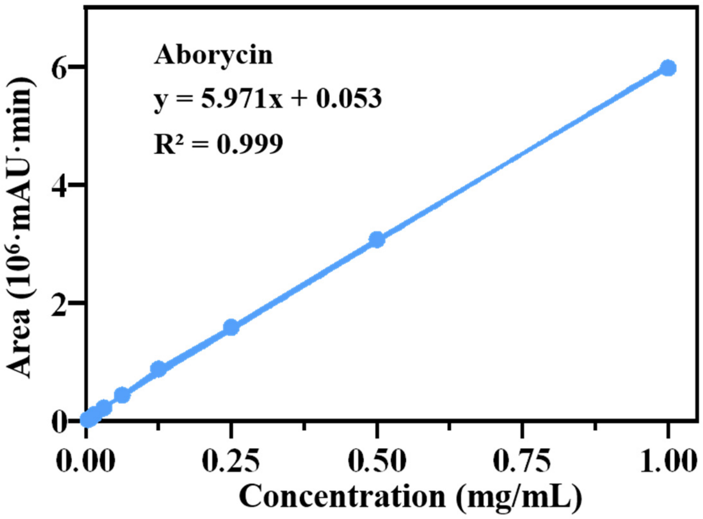


**Figure S15.** The standard curve depicting the relationship between aborycin concentrations and the HPLC peak areas.

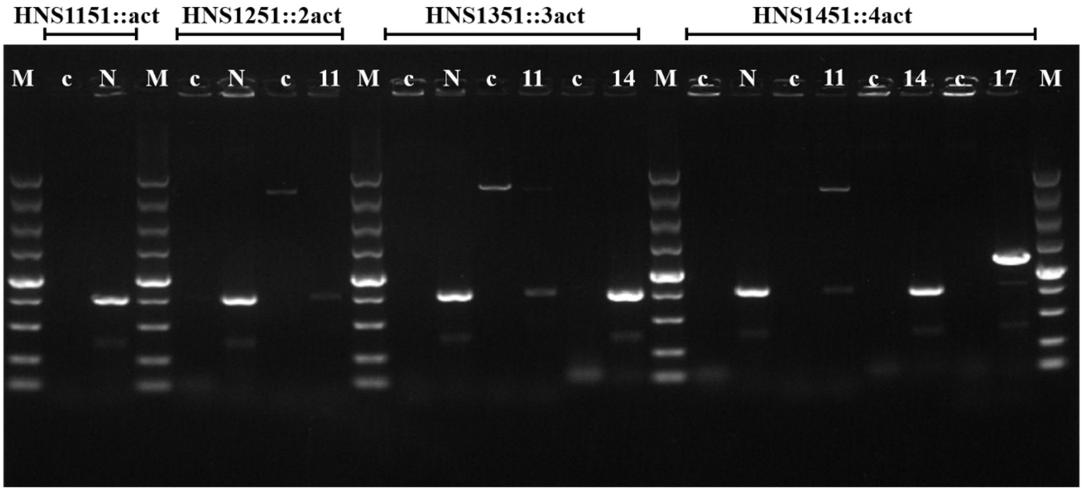


**Figure S16.** Identification of the plasmid pSET152::act introduced into the strains HNS1151-1451. M: 5 Kb DNA marker; c: negative control. N: native; 11: BGC11; 14: BGC14; 17: BGC17.

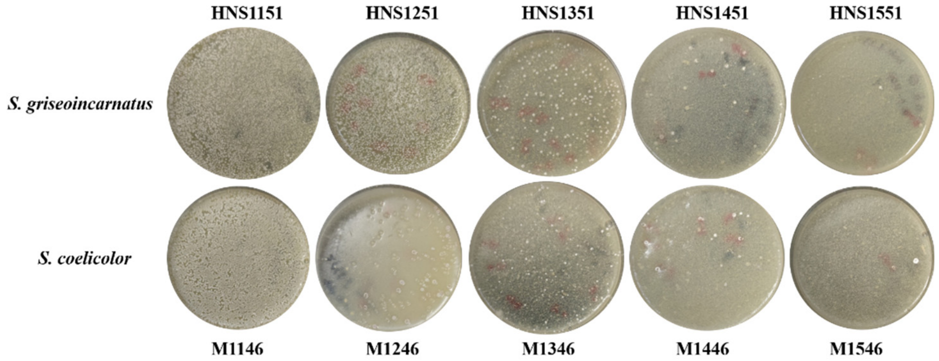


**Figure S17.** The conjugation effect of *attB* numbers on strains. Plasmid pSET152 was transferred from *E. coli* ET12567/pUZ8002 to various *Streptomyces* strains by intergeneric conjugation, and the conjugants were grown on apramycin (50 μg/mL) plates. The plates were incubated for 7 d and the number of colonies was counted. The results reveal a decrease in conjugation efficiency with an increase in the number of identical *attB* sites within the same strain.

**Table S1.** Basic genome sequencing features of HNS054

| Features | Chromosome |
|---|---|
| Sequencing technology | PacBio Sequel II, Illumina NovaSeq 6000 |
| Contig | 1 |
| Scaffold | 1 |
| Genome topology | Linear |
| Assembly size (bp) | 7523030 |
| GC content (%) | 72.3 |
| No. of ORFs | 6678 |
| No. of tRNA | 72 (45 species) |
| No. of rRNA (5S, 16S, 23S) | 18 |
| No. of BGCs | 21 |

**Table S2.** Endogenous CRISPR sequences in HNS054 genome

| Element | Id | Start | End | Spacer | Repeat consensus |
|---|---|---|---|---|---|
| CRIPSR | 1 | 794940 | 795069 | 1 | CAGCACGTGCAGAGGGCGCAGGAGTCCGTGCAC AGC |
| CRIPSR | 2 | 811402 | 811513 | 2 | CCACCGGGGCCGCCCGGACCGCCGG |
| CRIPSR | 3 | 935947 | 936046 | 1 | GGCTTCCACTGAGTGCGAGGGCTCCCGCCGAGTG GGCG |
| CRIPSR | 4 | 1081083 | 1081182 | 1 | GGGGCGCGGGGAACCGCGCGAACGCCCACCAC G |
| CRIPSR | 5 | 1209035 | 1209141 | 1 | GGGCGTTCACCCGCCGCCCACTGC |
| CRIPSR | 6 | 1512962 | 1513061 | 1 | GGTTGTTCGCGCAGTTCCCCGCGCCCCTGGGGGC |
| CRIPSR | 7 | 2004747 | 2004824 | 1 | GTCCAGCTCGGCGCGCAGCCGCTC |
| CRIPSR | 8 | 2282208 | 2282299 | 1 | TCGCGCAGTTCCCCGCGCCCCTG |
| CRIPSR | 9 | 2449899 | 2449991 | 1 | CGCGGGGAACTGCGCGACCAGCCAC |
| CRIPSR | 10 | 2505449 | 2505549 | 1 | GGGTCGTGGGCGGGCTGCTGGTGGCCGCC |
| CRIPSR | 11 | 2534316 | 2534420 | 1 | GTCAGCACCCACCGGTACAGCCAC |
| CRIPSR | 12 | 3292081 | 3292170 | 1 | TCGCGCAGTTCCCCGCGCCCCTTC |
| CRIPSR | 13 | 3772603 | 3772701 | 1 | GGCCGCGGCCGGCGGGTAGCCGTA |
| CRIPSR | 14 | 3853567 | 3853692 | 1 | GCGGGGCTCGTGGTCGGTCCCCGGGGGTTATGCC G |
| CRIPSR | 15 | 4517185 | 4517292 | 1 | CTCGGCGGCCTAGCTGCGCAGGCCAGGTC |
| CRIPSR | 16 | 4867640 | 4867699 | 1 | AGGGGCGCGGGGAACTGCGCGAC |
| CRIPSR | 17 | 5142849 | 5142939 | 1 | CGTCGCGCAGCCGCTCGGCCTCCT |
| CRIPSR | 18 | 5143275 | 5143365 | 1 | CGTCGCGCAGCCGCTCGGCCTCCT |
| CRIPSR | 19 | 5143494 | 5143583 | 1 | CGTCGCGCAGCCGCTCGGCCTCCT |
| CRIPSR | 20 | 5292916 | 5293019 | 1 | GGGGCGCGGGGAACTGCGCGAGCA |
| CRIPSR | 21 | 6004602 | 6004714 | 1 | GGCCGACGGCGTGCTCCGTGCCACACGGCCGC |
| CRIPSR | 22 | 6787654 | 6787745 | 1 | GTTCGCGCAGTTCCCCGCGCCCCTT |
| CRIPSR | 23 | 7066800 | 7066893 | 1 | AGGGGCGCGGGGAACTGCGCGCTC |
| CRIPSR | 24 | 7138726 | 7138844 | 1 | ACGGCCTGCCCGGTCCCGGCACCCTGGTCCGG |

**Table S3.** ANI and dDDH analysis of HNS054 to related species

| Strain | ANI (%) | dDDH (%) |
|---|---|---|
| *S. griseoincarnatus* RB7AG | 98.45 | 85.70 |
| *S. variabilis* JCM 4422 | 98.23 | 83.50 |
| *S. lusitanus* FZ202 | 95.28 | 59.60 |
| *S. griseorubens* JCM 4383 | 95.28 | 59.70 |
| *S. albogriseolus* LBX-2 | 94.17 | 53.90 |
| *S. fungicidicus* ATCC 21013 | 88.42 | 31.80 |
| *S. leeuwenhoekii* C34 | 86.71 | 26.90 |

**Table S4.** AntiSMASH-predicted BGCs in the HNS054 genome

| No. | Cluster No. | Type | Length (nt) | Most similar known cluster | Similarity (%) |
|---|---|---|---|---|---|
| 1 | BGC1 | Ectoine | 13753 | Ectoine | 100 |
| 2 | BGC2 | NRPS | 153463 | Naphthyridinomycin | 100 |
| 3 | BGC3 | RiPP-like | 10484 | Streptamidine | 75 |
| 4 | BGC4 | T3PKS | 41073 | Alkylresorcinol | 100 |
| 5 | BGC5 | Terpene | 22502 | Carotenoid | 54 |
| 6 | BGC6 | T2PKS | 72509 | Spore pigment | 83 |
| 7 | BGC7 | Ectoine | 8869 | Ectoine | 100 |
| 8 | BGC8 | NRPS | 32799 | Scleric acid | 17 |
| 9 | BGC9 | Siderophore | 10632 | Desferrioxamine B/E | 83 |
| 10 | BGC10 | Lanthipeptide-class-iv | 22672 | Venezuelin | 100 |
| 11 | BGC11 | Lasso peptide | 22493 | Aborycin | 100 |
| 12 | BGC12 | Phenazine | 20446 | – | – |
| 13 | BGC13 | Terpene | 20783 | Albaflavenone | 100 |
| 14 | BGC14 | Ripp-like | 11016 | – | – |
| 15 | BGC15 | Terpene | 19936 | Geosmin | 100 |
| 16 | BGC16 | Siderophore | 13239 | Grincamycin | 8 |
| 17 | BGC17 | Terpene | 26696 | Hopene | 92 |
| 18 | BGC18 | Ripp-like | 10215 | Informatipeptin | 57 |
| 19 | BGC19 | Ectoine | 15409 | Ectoine | 100 |
| 20 | BGC20 | NRPS-PKS | 100080 | Polyoxypeptin | 48 |
| 21 | BGC21 | Lanthipeptide-class-III | 22657 | SapB | 100 |

**Table S5.** Identification of natural *attB* sites in strains

| **Type of *attB* in SSR system** | **HNS054** | ***S. griseus* NBRC 13350 NC_010572** | ***S. coelicolor* A3(2) NC_003888.1** | ***S. albus* J1074 NC_020990** | ***S. atratus* SCSIO ZH16 NZ_CP027306.1** |
|---|---|---|---|---|---|
| φBT1 (*Sco*) | 59/62 (95%) | 46/48 (95%) | 62/62 (100%) | 38/41 (92%) | 57/62 (91%) |
| φC31 (*Sco*) | 47/51 (92%) | 46/51 (90%) | 51/51 (100%) | 48/51 (94%) | 47/51 (92%) |
| SV1 (*Sco*) | 54/55 (98%) | 47/52 (90%) | 55/55 (100%) | 18/18 (100%) | 18/18 (100%) |
| TG1 (*Sco*) | 33/33 (100%) | 28/30 (93%) | 46/46 (100%) | 31/33 (93%) | 43/46 (93%) |
| VWB1(*Sco*) | 51/51 (100%) | 53/53 (100%) | 53/53 (100%) | 53/53 (100%) | 50/50 (100%) |
| VWB2 (*Sco*) | 43/44 (97%) | 27/27 (100%) | 43/43 (100%) | 43/43 (100%) | 43/44 (97%) |
| VWB3 (*Sco*) | 38/39 (97%) | Not found | 27/27 (100%) | 27/27 (100%) | 27/27 (100%) |
| φJoe (*Sven*) | 34/38 (89%) | 27/27 (100%) | 26/26 (100%) | 33/36 (91%) | 38/38 (100%) |
| φK38-1 (*Sco*) | 47/48 (97%) | 50/55 (90%) | 61/61 (100%) | 30/32 (93%) | 35/38 (92%) |
| CBG73463 (*Sco*) | 54/57 (94%) | 16/16 (100%) | 57/57 (100%) | 15/15 (100%) | 15/15 (100%) |
| R4 (*Sco*) | Not found | 37/41 (90%) | 41/41 (100%) | 14/14 (100%) | 14/14 (100%) |

*Sco*: *S. coelicolor; Sven*: *S. venezuelae*

**Table S6.** The antibiotic sensitivity of HNS054

| Antibiotic | Concentration (μg/mL) | | | | | |
|---|---|---|---|---|---|---|
| | 0 | 12.5 | 25 | 50 | 75 | 100 |
| Ampicillin | ++ | + | + | + | + | + |
| Chloramphenicol | ++ | ++ | ++ | ++ | ++ | ++ |
| Kanamycin | ++ | + | + | − | − | − |
| Apramycin | ++ | + | + | − | − | − |
| Thiostrepton | ++ | ++ | ++ | ++ | ++ | ++ |
| Nalidixic acid | ++ | ++ | ++ | ++ | ++ | ++ |
| Tetracycline | ++ | + | − | − | − | − |
| | 50 | 100 | 150 | 200 | 250 | 300 |
| Spectinomycin | ++ | ++ | + | − | − | − |
| Hygromycin | + | − | − | − | − | − |

Note: −: not grown; +: weak growth; ++: normal growth

**Table S7.** Primers used in this study

| Primer | Sequence (5′-3′) | Description |
|---|---|---|
| Del-BGC11-up-fwd | ttttgagatctgaattccacATCCGTTGGCAAGGTTTGAT | Primers for the plasmid pKY01dB11 construction |
| Del-BGC11-up-rev | CCAAGGAGATCCTCACCGACTT | Primers for the plasmid pKY01dB11 construction |
| Del-BGC11-down-fwd | gtcggtgaggatctccttggACCCGAGACCTACGCATTACG | Primers for the plasmid pKY01dB11 construction |
| Del-BGC11-down-rev | acgacggccagtgccaagcttCGCAGCAGCCCTGTCCAC | Primers for the plasmid pKY01dB11 construction |
| ID-BGC11-fwd | gggaatccattggccttatgt | Primers validation of the BGC11 knockout in strain |
| ID-BGC11-rev | gaggagcttgtcactcatccg | Primers validation of the BGC11 knockout in strain |
| Del-BGC11-up-B-fwd | ttttgagatctgaattccacgtcccagacgagcgagatgcc | Primers for the plasmid pKY01dB11::attB construction |
| Del-BGC11-up-B-rev | aagggcacgccctggcacccgcaccgGTCCGTCGTCTCGGTCAGCAC | Primers for the plasmid pKY01dB11::attB construction |
| Del-BGC11-down-B-fwd | CGGTGCGGGTGCCAGGGCGTGCCCTTGGGCTCCCCGGGCGCGTACTCCACC CGCCAGCCACGACAGATG | Primers for the plasmid pKY01dB11::attB construction |
| Del-BGC11-down-B-rev | acgacggccagtgccaagctt GGTCGCCGCCCTCCTCACGC | Primers for the plasmid pKY01dB11::attB construction |
| BGC11-sgRNA-B-fwd | agtcctaggtataatactagtCGGGCAAGACTTAGTTTCATgttttagagctagaaatagca | Primers for the plasmid pKY01dB11::attB, pKY01dB11 construction |
| sgRNA-rev | gtggaattcagatctcaaaaa | Primers for the plasmid pKY01dB11::attB, pKY01dB11, pKY26dB17::attB, pKY44dB2::attB, pKY10dB14::attB construction |
| ID-BGC11-B-fwd | gtgccaggaccgccccacg | Primers validation of the BGC11 knockout and $attB_{\varphi C31}$ site introduction in strain |
| ID-BGC11-B-rev | gctgcgcggtgaggaccgg | Primers validation of the BGC11 knockout and $attB_{\varphi C31}$ site introduction in strain |
| Del-BGC17-up-fwd | ttttgagatctgaattccacCTGATGCCGTTCGTGCTG | Primers for the plasmid pKY26dB17::attB construction |
| Del-BGC17-up-rev | aagggcacgccctggcacccgcaccgCGTTCTACACTGCTGACCCAA | Primers for the plasmid pKY26dB17::attB construction |
| Del-BGC17-down-fwd | CGGTGCGGGTGCCAGGGCGTGCCCTTGGGCTCCCCGGGCGCGTACTCCACCAGGGAGTGACCACAGATGACC | Primers for the plasmid pKY26dB17::attB construction |
| Del-BGC17-down-rev | acgacggccagtgccaagcttTCGCCTCTTCGATCAGGGT | Primers for the plasmid pKY26dB17::attB construction |
| BGC17-sgRNA-fwd | agtcctaggtataatactagtCAGGCACGCAACTTCGCCTAgttttagagctagaaatagca | Primers for the plasmid pKY26dB17::attB construction |
| ID-BGC17-fwd | CCCGTGAGTGAGACTACGCA | Primers validation of the BGC17 knockout and $attB_{\varphi C31}$ site introduction in strain |
| ID-BGC17-rev | TCGGCTGGAAGTGGATGTC | Primers validation of the BGC17 knockout and $attB_{\varphi C31}$ site introduction in strain |
| Del-BGC2-up-fwd | ttttgagatctgaattccacCATGGAGAACATCACGTCGAAC | Primers for the plasmid pKY44dB2::attB construction |
| Del-BGC2-up-rev | aagggcacgccctggcacccgcaccgGCGGGAACCCAGTGAGCA | Primers for the plasmid pKY44dB2::attB construction |
| Del-BGC2-down-fwd | CGGTGCGGGTGCCAGGGCGTGCCCTTGGGCTCCCCGGGCGCGTACTCCACCCCAACATCAGAGCGATTACCG | Primers for the plasmid pKY44dB2::attB construction |
| Del-BGC2-down-rev | acgacggccagtgccaagcttCCGCTTGGTTGCCGTGTG | Primers for the plasmid pKY44dB2::attB construction |

| | | |
|---|---|---|
| BGC2-sgRNA-fwd | agtcctaggtataatactagtCGACGCTCCTCTCGTGGTCCgttttagagctagaaatagca | Primers for the plasmid pKY44dB2::attB construction |
| ID-BGC2-fwd | ccccgcgcgtccagcacac | Primers validation of the BGC2 knockout and *attB*$_{\varphi C31}$ site introduction in strain |
| ID-BGC2-rev | tgggccgtacggcggggttg | Primers validation of the BGC2 knockout and *attB*$_{\varphi C31}$ site introduction in strain |
| Del-BGC14-up-fwd | tttttgagatctgaattccacCTCGGAGGCCCGTATGCAC | Primers for the plasmid pKY10dB14::attB construction |
| Del-BGC14-up-rev | aagggcacgccctggcacccgcaccgGGCACTACACCTCGCTGAACAA | Primers for the plasmid pKY10dB14::attB construction |
| Del-BGC14-down-fwd | CGGTGCGGGTGCCAGGGCGTGCCCTTGGGCTCCCCGGGCGCGTACTCCACCGCAGCAGGGTGGTGAGGGT | Primers for the plasmid pKY10dB14::attB construction |
| Del-BGC14-down-rev | acgacggccagtgccaagcttAGGGCGTCGGGGTGGAACT | Primers for the plasmid pKY10dB14::attB construction |
| BGC14-sgRNA-fwd | agtcctaggtataatactagtCCGCCAGACGCTTCCAGCCCgttttagagctagaaatagca | Primers for the plasmid pKY10dB14::attB construction |
| ID-BGC14-fwd | gtcgcctccttctccctcg | Primers validation of the BGC14 knockout and *attB*$_{\varphi C31}$ site introduction in strain |
| ID-BGC14-rev | ggaacaagtgggcgtacacg | Primers validation of BGC14 knockout and *attB*$_{\varphi C31}$ site introduction in strain |
| pKCcas9sc-fwd | CCGGTCCAGTAATGACCTCAGA | Primers verification of pKY01dB11::attB, pKY26dB17::attB, pKY44dB2::attB, pKY10dB14::attB plasmid construction |
| pKCcas9sc-rev | TGCAAGGCGATTAAGTTGGGT | Primers verification of pKY01dB11::attB, pKY26dB17::attB, pKY44dB2::attB, pKY10dB14::attB plasmid construction |
| pSET152-fwd | GTCATAGCTGTTTCCTGTGTGAAATT | Linearized amplification of pSET152 |
| pSET152-rev | ACTGGCCGTCGTTTTACAACG | Linearized amplification of pSET152 |
| 054 gul-fwd | gttgtaaaacgacggccagtTCCGTTGGCAAGGTTTGATG | Amplification of aborycin BGC |
| 054 gul-rev | acacaggaaacagctatgacACGACGAGAAGGAGACCGAGG | Amplification of aborycin BGC |
| BGC11-fwd | TCCCGGGTGGTGCCGACGAC | Primers verification of pSET152::gul plasmid construction |
| BGC11-rev | GTCGGAACAGGAGAGCGCAC | Primers verification of pSET152::gul plasmid construction |
| BGC2 B-fwd | CCCTTCCTGGTTGGCTTGGTTT | Primers for *attB*$_{\varphi C31}$ integration detection |
| BGC2 B-rev | GCAGCCAGGCGAGTGGATTCTT | Primers for *attB*$_{\varphi C31}$ integration detection |
| BGC11 B-rev | ggcagctcctgcatcgactcg | Primers for *attB*$_{\varphi C31}$ integration detection |
| BGC17 B-rev | CCGACTTGACGGTGCCCTGGAAC | Primers for *attB*$_{\varphi C31}$ integration detection |
| BGC14 B-rev | CGCACCATCTCCTCTACAACACCACG | Primers for *attB*$_{\varphi C31}$ integration detection |
| ID-oriT-fwd | gcagagcaggattcccgttgagca | Primers for *attB*$_{\varphi C31}$ integration detection |
| Native B-rev | GGGGTGGCAGGAAGTTCAACGCTC | Primers for *attB*$_{\varphi C31}$ integration detection |

**Table S8.** Plasmids and strains used in this study

| Plasmid | Description | Source |
|---|---|---|
| pKCcas9dO | *ori$^{pUC}$, oriT, reppSG5, acc(3)IV, PJ23119(SpeI)-spacer-sgRNA-HR cassette, PtipA-SpCas9* | [36] |
| pKY01dB11 | pKY01dB11 with two homologous arms and the sgRNA transcription cassette for deleting the cluster BGC11 | This study |
| pKY01dB11::attB | pKY01dB11::attB with two homologous arms and the sgRNA transcription cassette for deleting the cluster BGC11 and introducing an artificial *$attB_{\varphi C31}$* | This study |
| pKY26dB17::attB | pKY26dB17::attB with two homologous arms and the sgRNA transcription cassette for deleting the cluster BGC17 and introducing an artificial *$attB_{\varphi C31}$* | This study |
| pKY10dB14::attB | pKY10dB14::attB with two homologous arms and the sgRNA transcription cassette for deleting the cluster BGC14 and introducing an artificial *$attB_{\varphi C31}$* | This study |
| pKY44dB2::attB | pKY44dB2::attB with two homologous arms and the sgRNA transcription cassette for deleting the cluster BGC2 and introducing an artificial *$attB_{\varphi C31}$* | This study |
| pSET152 | *oriT, $attP_{\varphi C31}$, $int_{\varphi C31}$, lacZa, ori$^{pUC}$, acc(3)IV* | [37] |
| pKC-gul | *acc(3)IV, ori$^{pUC}$, rep$^{pSG5}$, oriT, tipA-Scocas9,* j23119-gulspacer-gRNA, homologous region flanking *gul BGC* | In the laboratory |
| pSET152::gul | *oriT, $attP_{\varphi C31}$, $int_{\varphi C31}$, acc(3)IV, ori$^{pUC}$, gul BGC* | In the laboratory |
| pSET152::act | *oriT, $attP_{\varphi C31}$, $int_{\varphi C31}$, acc(3)IV, ori$^{pUC}$, act BGC* | [34] |
| **Strain** | **Description** | **Source** |
| *E. coli* DH5α | *F-φ80 lac ZΔM15 Δ(lacZYA-arg F) U169 endA1 recA1 hsdR17(rk-,mk+) supE44λ-thi-1 gyrA96 relA1 phoA* | [38] |
| *E. coli* ET12567(pUZ8002) | *dam, dcm, hsdS, cat, tet, tra, neo,* RP4 | [39] |
| *S. coelicolor* M1146 | *S. coelicolor* M145 (contains one native *$attB_{\varphi C31}$) Δact, Δred, Δcpk, Δcda* | [38] |
| *S. coelicolor* M1246 | *S. coelicolor* M1146 *Δcpk::$attB_{\varphi C31}$* | [27] |
| *S. coelicolor* M1346 | *S. coelicolor* M1246 *Δred::$attB_{\varphi C31}$* | [27] |
| *S. coelicolor* M1446 | *S. coelicolor* M1346 *Δcda::$attB_{\varphi C31}$* | [27] |
| *S. coelicolor* M1546 | *S. coelicolor* M1446 *Δact::$attB_{\varphi C31}$* | [27] |
| *S. coelicolor* M1346::3gul | *S. coelicolor* M1346 integrated three copy of pSET152::gul at the native *$attB_{\varphi C31}$* | [15] |
| *S. griseoincarnatus* HNS054 | Wide type strain, *S. griseoincarnatus* HNS054 with one copy of gul BGC at the native *$attB_{\varphi C31}$* | [17] |
| *S. griseoincarnatus* HNS1151 | *S. griseoincarnatus* HNS054 deleted BGC11 | This study |
| *S. griseoincarnatus* HNS1251 | *S. griseoincarnatus* HNS054 introduced X *$attB_{\varphi C31}$* at the adjacent loci of the deleted BGC11 | This study |
| *S. griseoincarnatus* HNS1351 | *S. griseoincarnatus* HNS054 introduced 2X *$attB_{\varphi C31}$* at the adjacent loci of the deleted BGC11 and BGC14 | This study |
| *S. griseoincarnatus* HNS1451 | *S. griseoincarnatus* HNS054 introduced 3X *$attB_{\varphi C31}$* at the adjacent loci of the deleted BGC11, BGC14 and BGC17 | This study |
| *S. griseoincarnatus* HNS1551 | *S. griseoincarnatus* HNS054 introduced 4X *$attB_{\varphi C31}$* at the adjacent loci of the deleted BGC11, BGC14, BGC17 and BGC2 | This study |
| *S. griseoincarnatus* HNS1151::gul | *S. griseoincarnatus* HNS1151 integrated one copy of pSET152::gul at the native *$attB_{\varphi C31}$* | This study |
| *S. griseoincarnatus* HNS054::pSET152 | *S. griseoincarnatus* HNS054 integrated one copy of pSET152 at the native *$attB_{\varphi C31}$* | This study |
| *S. griseoincarnatus* HNS1251::2gul | *S. griseoincarnatus* HNS1251 integrated two copy of pSET152::gul at the *$attB_{\varphi C31}$* | This study |
| *S. griseoincarnatus* HNS1351::3gul | *S. griseoincarnatus* HNS1351 integrated three copy of pSET152::gul at the *$attB_{\varphi C31}$* | This study |
| *S. griseoincarnatus* HNS1451::4gul | *S. griseoincarnatus* HNS1451 integrated four copy of pSET152::gul at the *$attB_{\varphi C31}$* | This study |

| | | |
|---|---|---|
| *S. griseoincarnatus* HNS1551::5gul | *S. griseoincarnatus* HNS1551 integrated five copy of pSET152::gul at the $attB_{\varphi C31}$ | This study |
| *S. griseoincarnatus* HNS1151::pSET152 | *S. griseoincarnatus* HNS1151 integrated one copy of pSET152 at the native $attB_{\varphi C31}$ | This study |
| *S. griseoincarnatus* HNS1151::act | *S. griseoincarnatus* HNS1151 integrated one copy of pSET152::act at the native $attB_{\varphi C31}$ | This study |
| *S. griseoincarnatus* HNS1251::2act | *S. griseoincarnatus* HNS1251 integrated two copy of pSET152::act at the $attB_{\varphi C31}$ | This study |
| *S. griseoincarnatus* HNS1351::3act | *S. griseoincarnatus* HNS1351 integrated three copy of pSET152::act at the $attB_{\varphi C31}$ | This study |
| *S. griseoincarnatus* HNS1451::4act | *S. griseoincarnatus* HNS1451 integrated four copy of pSET152::act at the $attB_{\varphi C31}$ | This study |
| *S. coelicolor* M1346::3act | *S. coelicolor* M1346 integrated three copy of pSET152::act at the $attB_{\varphi C31}$ | This study |

**Table S9.** Basic information of *Streptomyces* genomes

| No. | Strain | NCBI number | Size | Source |
|---|---|---|---|---|
| 1 | *S. griseoincarnatus* HNS054 | CP139576 | 7.52 | marine sponge |
| 2 | *S. violaceusniger* Tu 4113 | NC_015957.1 | 10.66 | soil |
| 3 | *S. bingchenggensis* BCW-1 | NC_016582.1 | 11.94 | soil |
| 4 | *S. griseus* NBRC 13350 | NC_010572.1 | 8.55 | soil |
| 5 | *S. avermitilis* MA-4680 | NC_003155.5 | 9.03 | soil |
| 6 | *S. scabiei* 87.22 | NC_013929.1 | 10.15 | plant-associated |
| 7 | *S. coelicolor* A3(2) | NZ_CP042324.1 | 8.67 | soil |
| 8 | *S. collinus* Tu 365 | NC_021985.1 | 8.27 | soil |
| 9 | *S. davaonensis* JCM4913 | NC_020504.1 | 9.47 | soil |
| 10 | *S. hygroscopicus* 5008 | NC_017765.1 | 10.15 | soil |
| 11 | *S. reticuli* TUE45 | LN997842.1 | 8.35 | soil |
| 12 | *S. globisporus* C-1027 | NZ_CP013738.1 | 7.61 | soil |
| 13 | *S. venezuelae* ATCC21782 | NZ_CP029190.1 | 7.53 | soil |
| 14 | *S. ambofaciens* ATCC 23877 | NZ_CP012382.1 | 8.30 | soil |
| 15 | *S. glaucescens* GLA.O | NZ_CP009438.1 | 7.45 | soil |
| 16 | *S. lividans* TK24 | NZ_CP009124.1 | 8.35 | soil |
| 17 | *S. vietnamensis* GIM4.0001 | NZ_CP010407.1 | 8.87 | soil |
| 18 | *S. leeuwenhoekii* C34 | NZ_LN831790.1 | 7.90 | soil |
| 19 | *S. xiamenensis* MCCC 1A01550 | NZ_CP009922.3 | 5.96 | mangrove |
| 20 | *S. noursei* DS30.6 | NZ_JAJUFB000000000.1 | 8.86 | soil |
| 21 | *S. lydicus* A02 | NZ_CP007699.2 | 9.30 | soil |
| 22 | *S. diacarni* LHW51701 | NZ_QOIN00000000.1 | 7.66 | marine sponge |
| 23 | *S. oceani* SCSIO 02100 | NZ_LJGU00000000.1 | 6.31 | marine |
| 24 | *S. reniochalinae* LHW50302 | NZ_QOIM00000000.1 | 7.69 | marine sponge |
| 25 | *S. microflavus* NA06532 | NZ_CP054926.1 | 7.79 | soil |
| 26 | *S. albus* DSM 41398 | NZ_CP010519.1 | 8.38 | soil |
| 27 | *S. violaceoruber* S21 | NZ_CP020570.1 | 7.92 | marine |
| 28 | *S. luteoverticillatus* CGMCC 15060 | NZ_CP034587.1 | 7.37 | marine |
| 29 | *S. formicae* KY5 | NZ_CP022685.1 | 9.61 | insect-associated |
| 30 | *S. niveus* SCSIO 3406 | NZ_CP018047.1 | 7.99 | marine |
| 31 | *S. spongiicola* HNM0071 | NZ_CP029254.1 | 7.18 | marine sponge |
| 32 | *S. atratus* SCSIO_ZH16 | NZ_CP027306.1 | 9.64 | marine |
| 33 | *S. pluripotens* MUSC 135 | NZ_CP021080.1 | 7.35 | mangrove |
| 34 | *S. aquilus* GGCR-6 | NZ_CP034463.1 | 10.39 | plant-associated |
| 35 | *S. indicus* CGMCC 4.5727 | NZ_FNFF00000000.1 | 8.23 | marine |
| 36 | *S. qinzhouensis* SSL-25 | NZ_CP042266.1 | 8.15 | mangrove |

| | | | | |
|---|---|---|---|---|
| 37 | *S. chattanoogensis* NRRL ISP-5002 | NZ_LGKG00000000.1 | 9.13 | soil |
| 38 | *S. kronopolitis* 6G-OA-10 | NZ_JAMFLE000000000.1 | 7.60 | plant-associated |
| 39 | *S. griseoincarnatus* RB7AG | NZ_JAMQBH000000000.1 | 7.71 | marine |
